# Transcription of Endogenous Retroviruses: Broad and Precise Mechanisms of Control

**DOI:** 10.3390/v16081312

**Published:** 2024-08-17

**Authors:** Abigail S. Jarosz, Julia V. Halo

**Affiliations:** 1 Science and Mathematics Division, Lorrain County Community College, Lorrain, OH 44035, USA; ajarosz@lorainccc.edu; 2 Department of Biological Sciences, Bowling Green State University, Bowling Green, OH 43403, USA

**Keywords:** endogenous retrovirus (ERV), long terminal repeat (LTR), co-evolution, KRAB-ZFP, chromatin, viral mimicry, embryogenesis, pluripotency, Dux

## Abstract

Endogenous retroviruses (ERVs) are the remnants of retroviral germline infections and are highly abundant in the genomes of vertebrates. At one time considered to be nothing more than inert ‘junk’ within genomes, ERVs have been tolerated within host genomes over vast timescales, and their study continues to reveal complex co-evolutionary histories within their respective host species. For example, multiple instances have been characterized of ERVs having been ‘borrowed’ for normal physiology, from single copies to ones involved in various regulatory networks such as innate immunity and during early development. Within the cell, the accessibility of ERVs is normally tightly controlled by epigenetic mechanisms such as DNA methylation or histone modifications. However, these silencing mechanisms of ERVs are reversible, and epigenetic alterations to the chromatin landscape can thus lead to their aberrant expression, as is observed in abnormal cellular environments such as in tumors. In this review, we focus on ERV transcriptional control and draw parallels and distinctions concerning the loss of regulation in disease, as well as their precise regulation in early development.

## 1. Introduction

**Figure 1:**
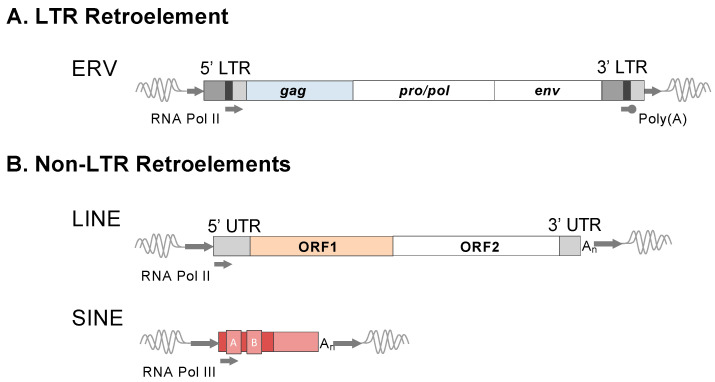
Structures and features of major retroelement types. Representations of canonical LTR and non-LTR retroelements are depicted. (**A**) Structure of a full-length ERV. Transcription signals are labeled in the LTRs for transcription initiated by RNA Polymerase II and Poly(A) stop signal. LTRs: U3, dark grey; R, black; U5, light grey. The minimal viral genes of an autonomous ERV are shown: *gag*, *pro*/*pol*, and *env*. All proviruses possess short 4-6 bp target site duplications (TSDs), as shown by the short flanking arrows. Non-autonomous ERV derivatives exist, such as those lacking *env* or *pol* and *env* (also refer to the main text). (**B**) Non-LTR retroelements include the long and short interspersed elements (LINE and SINE). A full-length retrotransposition competent LINE encodes two protein-coding open reading frames, ORF1 and ORF2, which, when translated, provide the necessary functions for mobilization. LINEs are autonomous elements that drive the retrotransposition of their own transcribed RNA intermediate or that from transcribed non-autonomous retroelements, including SINE. Therefore, non-LTR retroelements bear the hallmarks of LINE-mediated mobilization. LINE elements are transcribed by RNA Polymerase II and SINE by RNA Polymerase III. Due to distinct mechanisms of ERV and LINE integration, the TSDs of LINE-mobilized retroelements are of an average longer length (~15 bp), as depicted by the arrows flanking each element type.

Retroelements comprise a major class of transposable elements (TEs) that are characterized by mobilization involving the reverse transcription of an RNA intermediate transcribed from an existing element [1]. Reverse transcription of the intermediate results in a dsDNA that is then reintroduced into the genome at a unique position along a chromosome by integration. As the original element is left intact, this process is commonly referred to as a ‘copy and paste’ mechanism of amplification. Retroelements are further classified based on the presence or absence of long terminal repeats (LTRs) and are referred to as LTR and non-LTR retroelements (Figure 1A and Figure 1B, respectively) [1]. Under canonical conditions, non-LTR element spread is restricted to the cell in which they are mobilized, whereas LTR retroelements originate from the germline infection of exogenous retroviruses, and therefore, their ability to spread involves leaving the cell [2]. To avoid negative effects that could arise if retroelements were expressed, host cells have evolved several mechanisms to tightly control their transcription [3]. The ability to control these elements permitted their functional exaptation or ‘repurposing’ within the host genome, and retroelements have been recently characterized for their use in regulatory networks, such as innate immunity and during embryogenesis [4,5]. However, the deregulation of TEs is commonly observed in cancers and other diseases and can negatively impact the expression of local genes or promote oncogenic effects through various mechanisms [6,7]. In this review, we focus on recent advances from studies of ERVs concerning their transcriptional regulation in health and disease.

## 2. Endogenous Retroviruses

Retroviruses are positive-sense single-stranded RNA (ssRNA) viruses that have been infecting mammals and other vertebrates for hundreds of millions of years [8,9,10,11]. The retrovirus replication cycle is unique due to the hallmark requirement that, to establish a productive infection, the viral ssRNAs must be reverse transcribed to produce a double-stranded DNA (dsDNA) molecule that is then permanently integrated into the host cell’s genome [12]. Following integration, there is no mechanism of excision, and consequently, the integrated form is stably inherited as a genetic component of the cell and referred to as a provirus [12]. Due to the integration of the reverse-transcribed dsDNA molecule, infection of the germline (e.g., sperm or egg cells or during very early embryogenesis) leads to a provirus that has the potential to be transmitted vertically to offspring in a Mendelian fashion, referred to as an endogenous retrovirus (ERV) [2,13].

**Figure 2:**
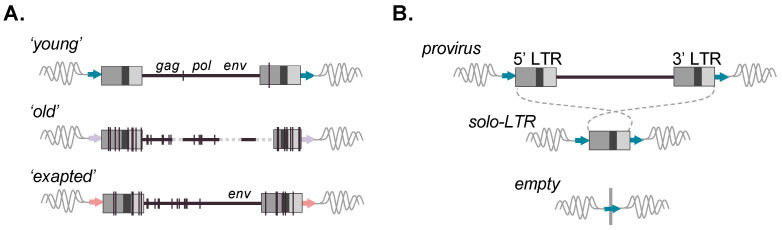
Evolution and allelic presence of ERV retroelements. (**A**) Full-length ERVs reflecting prototypical ages are depicted. Upper: ‘young’ ERV copy with little changes present; identical LTRs; and retained *gag*, *pol*, and *env* ORFs; Middle: ‘old’ ERV with many accumulated mutations, various deletions, and loss of gene coding capacity; Lower: ERV possessing an *env* ORF despite many proximal accumulated mutations and loss of other ORFs, indicative of retained coding function of the gene. Vertical lines represent mutations; dashed lines represent deleted proviral sequences. (**B**) Recombinational deletion results in the formation of a solo-LTR with the loss of the internal viral coding sequence but retention of the modular LTR form and its intrinsic sequence properties. Matched TSDs are likewise present following canonical solo-LTR formation (flanking arrows). (**B**) Possible alleles present for an ERV-derived locus post-integration. Upper: full-length; Middle: solo-LTR resulting from 5′–3′ LTR recombination. Lower: Prior to fixation of the insertion, a third ‘unoccupied’ allele can be present. ERV loci for which variable alleles are present within individuals of a host population are referred to as ‘insertionally polymorphic’.

At the time of integration, a canonical full-length ERV retains the characteristic properties of a replication competent integrated provirus [12]. Structurally, the ERV is comprised of a long directly repeated sequence located at either terminus, together representing the LTRs (5′ LTR and 3′ LTR), that flank an internal segment, including protein-coding genes required for replication (Figure 1A). Minimally, these include *gag*, *pro/pol*, and *env* [14]. Briefly, *gag* encodes structural proteins; *pro/pol* the enzymatic functions, including protease, reverse transcriptase, and integrase; and *env* the envelope surface glycoprotein that mediates receptor recognition and membrane fusion [14]. The internal 5′ untranslated region (UTR) upstream of *gag* houses a primer binding site (PBS) of sequence that is complementary to the cellular tRNA used to prime reverse transcription. Once integrated, the LTRs provide regulatory functions for the transcription and processing of spliced, as well as full-length, mRNAs that will ultimately be used as templates for protein synthesis or incorporated into budding virions [14]. In the absence of selection, mutations accumulate randomly at the neutral rate of the host, one that is markedly slowed from its exogenous replication [15]. Thus, ERVs provide a fossilized record of once (or still) infectious retroviral lineages. The majority of ERVs are ancient and have lost the ability to leave the cell due to accumulated mutations resulting in their decay [15]. However, some are observed to maintain intact genes due to benefits offered to the host or remain transcriptionally regulated despite replication incompetence. Several species’ genomes harbor ERV lineages with evidence of recent or ongoing germline invasion, as inferred by the presence of new copies (Figure 2A). These ‘young’ ERVs tend to bear close sequence homology to their exogenous source and may retain transcriptional activities or possess one or more open reading frames (ORFs). Recent studies have drawn attention to such lineages in felines [16,17], wolf-like canids [18,19], mule deer [20,21], bovines [22], and koalas [23,24,25].

Due to the mechanism of reverse transcription, the 5′ and 3′ LTR are identical in sequence at the time of integration and subsequently diverge [12]. Proviral LTRs are observed to undergo recombinational deletion, leading to the formation of a solitary LTR (solo-LTR) and resultant loss of the internal coding portion (Figure 2B). Therefore, a potential of three alleles may be present for a given insertion: a full-length provirus, solo-LTR, or (prior to fixation) the unoccupied site (Figure 2B) [26,27,28,29]. In general, solo-LTR formation tends to favor identical LTRs and thus appears to be inversely correlated with age [27]. However, deviations from this trend are observed, hinting that the pressures leading to solo-LTR formation are complex and likely to involve factors aside from sequence identity between the LTRs [28,30,31,32,33]. For a solo-LTR generated from identical pairs, the full nucleotide sequence should, in principle, be preserved, and the recombinant allele likewise retain the same potential for function. As with other repetitive elements, ERVs provide sources of genomic templates that can seed larger chromosomal rearrangements [34,35] or facilitate ectopic (non-allelic) gene conversion, resulting in the transfer of sequence information from highly similar but non-allelic ERV loci, thus influencing conversion ‘hotspots’ [26,34,36]. Well-characterized ERV-related hotspots are present within the human male-specific Y region (e.g., ERV1 LTR2 and LTR24 groups) [37]. ERV genes can also be subject to conversion, for example, the maintenance of the internal gene sequence as evidenced for ERV-V *env* (e.g., preservation of *ENVV1* in humans and simian primates) [38], as well as ERV-V *gag* (involving *gagV1* and *gagV3* in non-ape simian primates) [39].

Germline colonization followed by vertical passage has been a successful strategy for retroviruses [2,15]. For example, ERVs recognizably account for, respectively, 3.5 and 6% of the domestic dog and cat and 8 and 10% of the human and mouse reference genomes [40,41,42,43]. Upon their discovery, these elements were rightfully recognized as ‘viral fossils’ but often referred to as ‘junk DNA’ and widely assumed as inert [44]. Indeed, the repertoire of ERVs within a genome can be viewed as a limited but accessible record of once-infectious viruses ranging from the ancient to those still endogenizing a species [45]. Within this fossil record, the molecular signatures of past virus–host interactions may be gleaned, as well as subsequent co-evolutionary patterns between the two [2,45,46]. To say there are a growing number of exceptions to the ‘junk’ in our genomes is an understatement.

## 3. Nomenclature and General Properties of ERVs

### 3.1. ERV Nomenclature

Traditionally, ERVs have been principally classified by sequence homology of the *pol* gene with exogenous *Retroviridae* [47,48], which comprises two subfamilies (*Orthoretrovirinae* and *Spumaretrovirinae)* and 11 genera, according to the 2021 International Committee for Virus Taxonomy *[49]*. This classification scheme is further designated by one of three conventional classes: class I elements are similar to gamma- and epsilon-like retroviruses; class II are similar to alpha-, beta-, and delta-like retroviruses; class III are similar to the spuma-like retroviruses [50]. The nomenclature can be further adapted to notate ERVs by species presence using one or two letters (e.g., human ERV, HERV; *Canis familiaris*, CfERV), which may be accompanied by specification of the tRNA inferred to prime reverse transcription. For example, HERV-K members (class II, beta-like) have PBS sequence similarity to a tRNA^Lys^ [2]. These qualifiers are integrated into the RepBase classification of ERV/LTRs [51], which account for genomic presence by species [51,52]. Regarding ERVs, this classification is delineated by ‘superfamily’ (ERV1, ERV2, and ERV3; corresponding to class I, II, and III described above), followed by group, associated proviral sequence (‘-int’), and associated LTR [51,52]. For example, all human class II elements are beta-like; the youngest HERVs thus belong to ERV2 HML-2 HERV-K-int LTR5Hs [51]. Further discrimination of ERV loci by chromosomal location is by cytoband (e.g., HERV-K 11p15.4) [53,54]. A proposal of nomenclature using a systematic approach incorporates element type, locus-specific information, and species annotation as a system to account for orthologs between species, as well as insertionally polymorphic loci [47]. Given the growing number of identified ERVs over time [3], the challenges of adopting such a revised if common system are obvious.

### 3.2. General Properties and Recent Findings of Select ERV Groups

The estimated times of germline colonization between recognizably retroviral derived ERV groups is tremendous. The oldest ERVs in the human genome belong to the ERV3 spuma-like ERV-L that entered the germline >70 mya [55]. Subsequent amplification waves were in simian primates until extinction ~40–30 mya (HERV-L, e.g., MLT2s) and in mice (MERV-L, e.g., MT2s) around ~10 mya and again ~2 mya [56]. The related ERVL-MaLR are among the most abundant ERVs in humans and mice and share sequence homology with ERV-L *gag*, suggesting a distant common precursor [57]. ERVL-Mal-R insertions in the human genome that predate the human–mouse split (e.g., MLT1s) were later amplified in primates (e.g., MSTs) and simian primates (e.g., THE1s) [58]. All known ERV-L are *env*-less and ERVL-MaLR *pol*- and *env*-less, suggesting intracellular spread [59]. Interestingly, a transcriptionally competent ERVL-MaLR copy (THE1D) on human chr7 is predicted to possess a full-length (464 aa) ORF, but any function remains unknown [60]. Members of ERV-L (e.g., MLT2s) and ERVL-MaLR (e.g., MLT1s) are also present in dogs [42], as well as elephants [61], but are absent in opossums [58], suggesting propagation of ERV-L and ERV-MaLR in the eutherian ancestor ~110 mya [62].

The ERV2 beta-like HERV-K entered the germline ~55 mya prior to the New/Old World monkey (OWM) split [63], later followed by the HML-2 lineage ~35–30 mya in OWMs, as well as distinct HML groups (e.g., HML-1 to -11) [54]. HML-2 continued to invade germlines of primates’ ancestors; its most recent infectious derivative, HML-2 LTR5Hs, accounts for ~150 human-specific insertions, of which ~36 are unfixed in humans [64,65]. The youngest provirus may have integrated within the last ~100,000 years, suggesting activity in anatomically modern humans [64]. HML-2 infected gorillas more recently, as evidenced from ~150 species-specific proviruses, many of which are unfixed with identical LTRs [66]. LTR5Hs is the only HERV group that has proviruses with all ORFs [64,67]. Though no naturally occurring provirus is infectious, two inferred progenitors are: *Phoenix* [68] and HERV-K_CON_ [69]. HERV-K members were frequently subject to recombinational deletion, as >90% of the annotated copies are in the solo-LTR form [54,64].

One of the most abundant ERVs in human genomes is the ERV1 gamma-like HERV-H that entered the germline ~40–25 mya prior to the New/Old World monkey split and then was amplified mostly in OWMs [70]. Subsequent waves of propagation over timeframes ~20–9 mya and ~10–4 mya drove expansions of *env*-deficient copies [32,70,71,72]. As is reflected in RepBase, HERV-H LTRs are traditionally classified into four subgroups (LTR7, 7b, 7c, and 7y); their recent phylogenetic refinement identifies eight previously unrecognized ones, the youngest from the proposed classifications of 7up1/2, 7u, and 7y copies (reported in Dfam) [72]. The refined analysis divulges a dynamic recombination-driven history of HERV-H LTRs involving the gain, loss, and exchange of *cis*-regulatory functions contributing to subgroup-specific functional motifs [72]. HERV-H is notable for a shift in allelic presence from most ERV groups, in which proviruses account for >60% of all loci [28,31,33]. Though an explanation is not entirely clear, this shift in provirus presence hints at selective constraints of internal sequence properties [30,31,32].

The ERV1 gamma-like HERV-W (LTR17) lineage entered the germline prior to the New/Old World monkey split ~40 mya and later infected OWMs between ~35 and 25 mya [53]. It was spread simultaneously via intracellular retrotransposition, evidenced by copies that lack LTRs and possess polyA tails, along with longer TSDs at consensus targets of LINE-1 ORF2p [73]. HERV-W was originally studied for a possible role in neurodegenerative diseases [74,75]. Characterization of the lineage led to the remarkable finding of an exapted role of the *env* of one such member in placentation: *ERVWE1* (*syncytin-1*; 7q21.2) [76,77], noteworthy for belonging to the RD114/D-type interference group that utilizes the *ASCT2* receptor [78]. Significantly, *ERVWE1* is one of a handful of ERVs with an *env* gene independently exapted for analogous functions over placental mammals by convergent evolution [76]. A *cis*-acting motif recently identified within the 3′ UTR of *syncytin-1* enhances *env* expression via currently unknown interactors (‘syncytin post-transcriptional regulatory element’; SPRE) [79,80]. Interestingly, functional SPRE-like motifs appear to be present in other syncytins (e.g., macaque *mac-syncytin-3*, dog *syncytin-Car1*, and tenrec *syncytin-Ten1*), as well a variety of unrelated human ERV1 lineages, most abundantly HERV-W/LTR17 (~40 loci) and HERV9/LTR12 (~30 loci) [80]. SPRE functions aiding in the post-transcriptional regulation of lineage-specific ERV groups would be a significant finding [80].

## 4. Regulatory Features of ERVs

**Figure 3:**
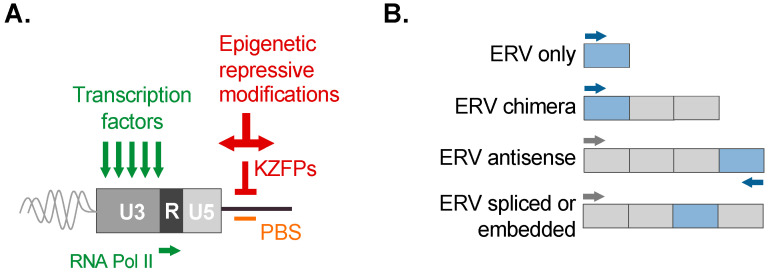
Overview of ERV control and ERV-associated transcripts. (**A**) ERV LTRs possess intrinsic features for transcriptional activity that can promote their expression and use as promoters or enhancers, such as transcription factor binding sites, as well as transcriptional signals recognized by RNA Polymerase II (summarized in green). Silencing of ERVs is achieved via epigenetic repressive modifications, including histone modifications and DNA methylation. A potent mechanism of silencing is the binding of the ERV primer binding site (PBS; labeled in orange) used during reverse transcription. Repressive binding of the PBS is mediated by a member of the Krüppel-associated box zinc finger protein family (KZFP; labeled in red). KZFP subsequently scaffolds epigenetic silencing complexes to exert potent silencing and promote heterochromatin spreading (summarized in red). The modular nature of an ERV LTR is depicted showing the unique (U3 and U5) and repeat (R) segments. (**B**) Examples of ERV-associated transcripts observed in tissues (also refer to the main text).

ERVs exert dramatic influence on the transcriptional landscape as well as the evolutionary shaping of the host genome. Many members of ERV lineages have retained biological properties and have been ‘borrowed’ for a benefit offered to the host, in which they are regulated (Figure 3A). In particular, the LTRs possess regulatory features for transcription by cellular machinery and can therefore act as promoters or long-range enhancers of host genes [13]. Likewise, host species possess repressive mechanisms to recognize ERVs and exert control over their activation [3]. Importantly, the potential of an ERV to be expressed is not limited to LTR-driven transcriptional mechanisms. LTRs may also be embedded within transcripts by readthrough from the transcription of alternate promoters of conventional genes (or even other LTRs) or can be spliced into mRNAs along with the functional sequence (Figure 3B). lncRNAs too were previously thought to have no biological function, and growing evidence implicates the functional relevance of lncRNAs, including those associated with ERVs [81,82,83,84,85]. Owing to these collective properties, ERVs are now recognized as a major force of regulatory innovation [5,29,86].

### 4.1. ERV LTRs Are Enriched in Transcription Factor Binding Sites

All retroviral LTRs, and, hence, those sourced from an ERV, possess a modular structure of unique segments U3 and U5 that are separated by a repeat segment R (5′ U3–R–U5 3′) (Figure 3A). Within these segments are regulatory *cis*-acting sequences corresponding to transcription factor (TF) binding sites (TFBSs) and the RNA Pol II TATA-box-like core promoter (usually in the U3) and a polyadenylation signal (usually in the R) [87]. Of note, the presence, placement, and sequence of these motifs can vary widely across ERV lineages [88,89].

LTRs are highly enriched for TFBSs or combinations thereof, implicating ERV propagation results in the deposition of not only of canonical promoters but also of directly associated *cis*-acting regulatory sequences. Curation of a TFBS presence within ERVs implies the functional evolution of such sites. For example, an analysis of ENCODE TFBS profiles from 13 human primary cell lines found roughly 15% overlap with LTRs, of which there was 8% overlap within 10kb of a predicted gene transcription start site (TSS) [90]. An analysis of ENCODE and Roadmap Epigenomics ChIPseq data for 97 TFs identified 794,972 ERV-encoded TFBSs over the human genome [86]. These can be parsed into clusters involved in shared regulatory functions, as inferred by the presence of common TFBSs (i.e., HERV/LTR shared regulatory element or HSRE). In this study, the authors identified eight such HSREs and their differential presence over ERV LTR groups, for example, the pluripotency cluster TFBSs Sox2, OCT4, and NANOG; embryonic endoderm cluster TFs GATA4/6, Sox17, and FOXA1/2; B-lymphocyte cluster TFs PAX5 and PBX3; and the chromatin architecture TF CTCF; many general TFBSs are present as well [86]. Importantly, a HSRE presence is not fully consistent with ERV phylogenetic classifications, and HSREs are instead differentially enriched within LTRs from distinct groups [86]. Younger ERV groups (e.g., LTR7 members, LTR5Hs, LTR6A, and MER11C) tend to have more pluripotent TFBSs; these TFBSs are rarely observed in exogenous viruses [86]. Generally, young LTRs tend to be CpG-rich, and CpG-rich LTRs tend to be bound by transcription initiation-associated TFs than CpG-depleted ones [91]. Long term, CpG sites are inevitably lost due to deamination and other mutations [91]. LTRs from older groups are overrepresented in enhancer regions compared to younger groups, suggesting the likelihood of an element to serve a regulatory function increases with age [91]. Based on data of chromatin accessibility and modification, a recent analysis of ENCODE data identified >924,000 candidate *cis*-regulatory elements (cCREs) in the human genome [92], of which 10.2% are primate-specific based on a comparison of 241 genomes of placental mammals of the Zoonomia Project [93]; 90% of these cCREs overlap TEs, of which 34.9% are within LTRs [92,93]. Thus, LTRs may account for around one-third (and TEs may account for nearly all) of primate-specific *cis*-regulatory elements. A subsequent study of 367 TFs identified ~15.6 million TFBSs using ChIPseq data of 785 cell and tissue types, of which 24.5% are primate-specific; 86.1% of these TFBSs overlap TEs, of which 22.4% are in LTRs [93]. Thus, a significant potential for regulatory innovation in primates appears to lie in ERVs and other TEs. It is important to remember that mutations post-insertion may impact the functional potential of LTR use, for example, by altering TFBS motifs or methylation sites. Such changes are subject to drift or other modes of selection and thus may vary in presence among individuals within a population. A population genetics approach is offered from the analysis of unique TFBSs present in the 5′ LTRs of HERV-K proviruses using the 1000 Genomes Project data [94].

### 4.2. LTRs Provide a Source of Modularity to Gene Regulation

Given their intrinsic properties, LTRs have indeed been utilized in mammalian evolution for transcriptional promoter and enhancer functions [95]. Additionally, the tendency to recombine neatly to the solo-LTR form introduces essentially finished promoters in modular form to new genomic locales. For example, LINE-1 retrotransposition is also driven by RNA Pol II from a 5′ internal core promoter; however, most new LINE-1 insertions are 5′ truncated and therefore incapable of conferring similar *cis*-regulatory functions [96,97]. Over evolutionary scales, propagation waves of lineage-specific ERVs thus dispensed numerous modules of functional potential that have fueled innovation in the regulation of genes or gene networks. Recent developments in ‘omics’-based techniques enable the direct interrogation of genetic and epigenetic alterations throughout a given cell or tissue type of interest. Importantly, these studies continue to reveal a history of virus–host co-evolution that is deeply intertwined and elegantly complex. The mechanisms of ERV-mediated regulation of transcriptional networks in immune defense were exemplified in a 2016 landmark study by Chuong et al. [4]. In that study, the authors showed that the propagation of lineage-specific γ-like ERVs (e.g., ERV1 MER41s) dispensed a reservoir of IFNγ-inducible LTR enhancers of multiple immune-related genes throughout the genome [4]. MER41Bs were discovered to be enriched for STAT1 binding, and one was identified as solely responsible for driving the expression of *AIM2*, a cytosolic foreign DNA sensor that activates the inflammatory response [4]. In addition to innate immunity, the regulatory exaptation of ERVs has been documented in processes including embryogenesis [98], placentation [99], and the evolution of regulatory differences between species [100,101]. Conversely, the activation of normally repressed ERVs can affect cancer initiation and progression in a unique phenomenon referred to as ‘onco-exaptation’, for example, by providing promoters of proto-oncogenes or of alternate oncogenic isoforms [6,7,102,103,104,105,106].

### 4.3. ERVs Are Regulated by Epigenetic Control

The necessity of strict ERV regulation to avoid the aberrant activation of local genes and counter the threat of insertional mutagenesis is obvious. As will be discussed later in Section 6, many ERVs are activated in very early cellular development, in which the genome is hypomethylated and accessible; these ERVs are rapidly silenced during differentiation and, in principle, remain tightly regulated in normal somatic tissues [3]. Silencing is enforced via multiple mechanisms, including histone modifications and DNA methylation, leading to a repressive heterochromatic state in what has been referred to as an ‘epigenetic corset’ [107].

In both mice and humans, targeting the ERV PBS for silencing is a potent strategy that is principally facilitated by KRAB-ZFPs (KZFPs) (Figure 3A). Functionally, members of the KZFPs contain at least one N-terminal Krüppel-associated box (KRAB, a motif related to the ~620 my old PRDM9/Meisetz, a determinant of recombination hotspots in meiosis [108,109]) and a C-terminal array of Cys_2_-His_2_ (C2H2) DNA-binding zinc-finger protein (ZFP, or ZNF) domains [110]. During silencing, the ZFP binding to an ERV recruits the co-repressor and ‘master regulator’ of canonical silencing TRIM28 (or KAP1) to bind the KRAB domain. This complex serves to scaffold heterochromatin-inducing factors as the H3K9 methyltransferases (e.g., SETDB1 and SUV39h), deacetylase complexes (e.g., NuRD), and HP1 to exert potent repression [106]. This manner of direct KZFP repression is bypassed for solo-LTRs, perhaps providing a selective context for solo-LTR formation or exaptation for tissue-specific regulation [29]. Sumoylation of TRIM28 or the actions of other chromatin remodeling factors enhances its localization to ERVs [111]. TRIM28 repression can act as a methylation ‘hub’ that can promote heterochromatin spreading to the surrounding genome, as facilitated by HP1 recruitment of SETDB1, as well as other H3K9-specific methyltransferases [3,110]. The HUSH complex recruits the chromatin modeler MORC2 and SETDB1 for H3K9me3 deposition; it represses HIV-1, as well as young ERVs and LINE elements [112]. KZFPs involved in ERV silencing also include H3K9me3-independent marks [113]. The deposition of repressive histone marks targets sites for rapid and stable de novo CpG DNA methylation by DNMT1, DNMT3A, and DNMT3B, generally considered to serve as an epigenetic ‘switch’ to maintain LTR silencing in differentiated tissues [3]. A general correlation of element age and methylation status indicates younger (i.e., CpG-rich) ERVs tend to be DNA methylated and, thus, more susceptible to reactivation by DNA methylation inhibitors (DNMTis), a phenotype that is synergistically enhanced by the knockdown of H3K9 methyltransferases (HMTs, e.g., SETDB1, SUV39h, or EZH2), whereas ones of an intermediate age tend to bear repressive histone marks, particularly H3K9me3, and are more sensitive to the knockdown of HMTs [114]. Most of the oldest LTRs (i.e., CpG-poor, e.g., older ERV-L, Gypsy elements) appear susceptible to neither DNMTis nor the knockdown of HMTs, indicating their transcriptional inactivation due to loss-of-function mutations [114]. However, as will be discussed, it is noteworthy that ERV-L-associated transcripts are observed in many human tumors, as well as during embryogenesis, and therefore such loss-of-function does not appear to generally apply to ERV-L group-wide. The susceptibilities of ERVs to DNMTis or HMTs differ between cell lines, which implies that differential expression resulting from deregulation of these pathways is likely to be reflected in tissues [114].

The KZFPs are notable as the largest family of ZFP transcriptional regulators in humans and mice and emerged in the Sarcopterygian ancestor of tetrapods, lung fish, and coelacanths ~420 mya [3,110]. Of note, its emergence follows the phylogenetically supported marine origin of the oldest known ERVs, of the class I spuma-like foamy retroviruses, >450 mya around the origin of jawed vertebrates [8]. Later in eutherians, as waves of ERVs propagated ancestral germlines, KZFPs rapidly expanded and diversified in response, resulting in respective species’ copy numbers in the hundreds, with evidence of selection at the C2H2-binding domains [115,116]. Most species analyzed have 200–400 copies; mice have nearly 700 [115]. Humans possess at least 378 KZFPs; over one-third are the products of recent duplications and restricted to primates [109], and over two-thirds have a TE as the primary target [117]. KZFPs also tend to be of evolutionarily similar ages to the ERVs they silence, with the youngest possessing the highest affinities for TRIM28 [116]. On the other hand, nearly all ancient KZFPs are inefficient recruiters of TRIM28 but appear to be selectively constrained, suggesting alternate functions [117]. Considering the genome-wide TFBS presence in humans, motifs corresponding to KZFP-binding sites have the highest enrichments in ERVs (as well as other TEs) [93]. Among outliers of the most TFBSs overlapping ERVs [93] are KZFPs implicated in H3K9me3-mediated silencing (ZNF586 and ZNF680), as well as H3K9me3-independent LTR silencing (ZNF329 and ZNF331) during early development [113]. ZNF350 (or ZBRK1), ZNF418, and ZNF134 are also identified [93]. KZFP expansion has been suggested as a host mechanism to prevent ERV spread as part of an evolutionary ‘arms race’, in which the genetic escape of KZFP-repressive binding of an ERV selects for emergent altered KZFPs and cycles back and forth [115]. However, particularly in the case of ERVs, the KZFPs’ targets comprise a vast majority (and perhaps all) of elements technically no longer capable of infection –but that nonetheless retain the ability to be transcriptionally used if regulated. ERV/KZFP interactions are widely implicated in establishing species-specific networks in early development, and many KZFP sites are bound by tissue-specific TFs and display characteristics of enhancers at later stages and in adult tissues [115]. For example, the primate-specific KZFPs ZNF417 and ZNF587 repress HERV-K members in embryonic stem cells and later maintain control of the ERVs in the developing and adult human brain [118]. Alterations of distinct KZFP/TE profiles are observed during brain development, in which they serve as alternate promoters of neurogenesis-specific genes [119]. Thus, an arms race alone is insufficient to explain the selection and maintenance of KZFPs [115]. Alternatively, the regulatory use of ERVs by KZFPs is proposed to promote their domestication and drive key aspects of species evolution and transcriptional nrtworks [115,116,120].

### 4.4. ERV Silencing Mechanisms Are Reversible

The loss of tight epigenetic control likewise features the disruption of ERV/LTR regulation normally silenced to promote genomic stability, which is associated with several aberrant pathologies [7,46,121,122]. Extensive chromatin remodeling occurs during malignant transformation, resulting in the redistribution of DNA methylation across the genome and accompanied accessibility of ERVs and other retroelements [123,124]. Hypomethylation is a hallmark characteristic of tumors and is recapitulated in cell models of cancer [125,126]. For example, constitutive signaling by Ras oncogenic overexpression leads to hypomethylation in a variety of cellular models of transformation, and while minimally expressed in hTERT immortalized cells, ERVs are highly transcribed in Ras-transformed cells [94,127,128,129]. Loss of repressive histone marks is accompanied by the aberrant expression of ERVs [123]. As discussed in Section 5, the alteration of both epigenetic properties contributing to expressed ERVs (and the consequences of their expression) has been of increasing interest to the field regarding tumor immunogenicity and immunotherapy [130,131,132,133]. Importantly, beyond a loss of repressive silencing, relevant LTR-specific changes alter TFBSs and therefore the potential for silencing, as well as transcriptional use of those LTRs [94]. The properties contributing to ERV expression thus converge on themes regarding direct LTR regulation (i.e., TSS in the LTR) that are dependent on (i) the differential access of LTRs as promoters given a particular cell state, (ii) the differential presence of TFs specific to accessible LTRs, and (iii) underlying genetic variations that are intrinsic to the LTRs themselves. The silencing of most ERVs implies their expression is intrinsically tied to their accessibility within chromatin, as well as the ability to be recognized. Given observations of differential ERV activation upon treatments with DNMTis (resulting in the tendency of ‘younger’ age ERVs to be expressed) or HMT inhibitors (expression of ‘intermediate’ age ERVs) [114], the prediction can be made that the internal inclusion of ERVs within transcripts may tend to originate from passive transcriptional effects, particularly regarding older integrants.

## 5. ERVs Are Derepressed in Abnormal Cellular States

### 5.1. ERV Expression Is Associated with Human Disease

The discovery of ‘RNA tumor virus’-like sequences in human DNA sparked decades of research seeking connections to cancer [134,135]. The sequencing of the human genome, and, later, whole genomes of individuals, expedited the identification and characterization of a multitude of ERVs [3]. ERV expression in the form of elevated mRNAs and ERV-encoded proteins is now known to occur in tumors and cell lines that model tumors and other environments. For example, transcripts of HERV-H, HERV-K, HERV-F, HERV-R, and HERV-S have all been observed in various cancer cell lines [136]. HERV-K HML-2 expression is correlated with cancers, including breast cancer, ovarian cancer, germ cell tumors, prostate cancer, melanoma, lung cancer, lymphoma, and others [2,7,137,138]. HML-2 LTR activation can aberrantly regulate nearby genes associated with breast cancer [139]. HERV-W expression is correlated with multiple sclerosis (MS), bipolar disorder, and schizophrenia [140,141,142]. HERV-H transcripts are significantly elevated in head and neck cancers, and HERV-E and HERV-K HML-6 are significantly downregulated in the same samples [143]. HERV-H drives many lncRNAs associated with various cancers, such as teratocarcinoma, bladder carcinoma, testicular tumors, and others [7]. ERV products display oncogenic properties, for example, the HERV-K proteins Rec and Np9 (respectively, from spliced mRNAs from type II and type I HML-2 proviruses) [144,145]. The Env proteins of HERV-K, HERV-H, and others possess immunosuppressive properties, suggesting an ability to modulate the immune response [146,147], as well as potential vaccine targets [148]. HERV-K Env can induce TFs in pathways associated with oncogenic transformation [149], as well as elicit cytokine release [150]. HERV-W Env has been identified in neural plaques of MS patients and contributes to the cellular damage of axons in MS [151,152] as well as cell-cell fusion in some cancers [153,154]. This Env has also been shown to induce IFN-ß innate immune signaling, leading to neuronal apoptosis in early-onset schizophrenia [155]. Collectively, these and other similar observations continue to motivate research seeking to determine the scope of ERV involvement in disease, with obvious interest in establishing meaningful links to phenotypes. It is important to keep in mind that the deregulation of other retroelement types (e.g., LINE and SINE; Figure 1B) can drive aberrant phenotypes, including oncogenic mutagenesis [156]. Also of importance, ERVs are expressed in healthy tissues in humans and animal models [19,157,158,159,160].

### 5.2. ERVs Are Broadly Expressed in Various Cell Types

Within the past decade, the sequencing of whole transcriptomes facilitated the discovery that ERVs are expressed in every examined tissue and cell line [160]. These findings beg questions of which ERVs are expressed and in which cell types. Though earlier studies mostly focused on members of particular ERV groups (e.g., HERV-K and HERV-W) or were limited to reported expressed ERVs according to broad classifications (e.g., ‘ERV1’ and ‘ERV-L’), it is now understood that there is a high degree of heterogeneity of expressed ERVs that differ vastly in representation by cell type [130,137,158]. In fact, thousands of transcribed ERVs are observed. Analysis of GTEx RNAseq data across normal tissues suggests some 13,889 ERVs are expressed, contributing to 0.19–1.9% of polyA RNAs across 42 tissue types [158]. Such targeted approaches to identify individually expressed ERVs also pinpoint exact expressed loci in cancers. For example, an analysis of prostate, breast, and colon cancer TCGA RNAseq identifies numerous differentially expressed ERV loci, and the top up- and downregulated loci differ strikingly in all three cancer types (two exceptions are the upregulated HERVs at 19q13.12a in breast and prostate tumors and HERV-L at 8q24.3d in breast and colon tumors) [161]. Though the significance is not clear, the two top upregulated prostate cancer ERVs are situated in a chr22 region that has been linked to chromosomal rearrangements HERV-K11 LTR5Hs 22q11.21 and HERV-K HML-2 LTR5B 22q11.23 [161]. This latter provirus is notable for control by a ~550 bp upstream solo-LTR5H, which has been characterized to drive the spliced lncRNA of LTR5Hs-B22q11.23, *PCAT14*, a prostate cancer biomarker of unknown function [162,163]. A recent study revealed the solo-LTR possesses nearly 50 TFBSs (nearly half of which correspond to ZNF-binding motifs) that are absent from related LTR5H members [157]. The unique TFBSs include a PRDM9 motif [157]; normally solely restricted to germ cells, PRDM9 is aberrantly expressed in some cancers, including prostate, and structural variant breakpoints frequently neighbor the TFBS motif [164]. Though speculative, the LTR has been implicated in an oncogenic translocation in the form of an overexpressed LTR_Hs-B-*ETV1* fusion transcript in a prostate tumor of an *ETV1*-truncated variant [165]. Recent studies have taken further advantage of RNAseq to infer ERV-sourced chimeric transcripts (i.e., possessing the ERV-derived sequence, as well as exonic sequence, of a conventional gene) (Figure 3B) as an indication of *cis*-regulatory transcriptional activities associated with ERV expression [127,161,166]. The findings revealed expressed ERVs in HRAS-transformed cells contribute to transcripts associated with standalone LTRs (i.e., ERV-only sequence with apparent TSSs in the LTR), as well as ones predicted to be LTR-initiated chimeras of genes or lncRNAs [127]. About 40 ERV-associated locus-specific transcripts from HRAS-transformed cells were also identified within TCGA RNAseq from breast, colon, or prostate tumors (e.g., including members of HERV-L, HERV-FRD-like PABL_A, and HERV-H) [127]. These findings suggest the presence of locus-specific changes controlling ERV expression that may be recapitulated in certain cell types. Such changes may correlate with LTRs expressed upon activation of common signaling pathways, but ERV expression is not precisely coordinated within perturbed cellular states.

### 5.3. The Cancer ERV Transcriptome Is Limited but Complex

An understanding of the larger scope of the potential impact of ERV expression is aided by the deeper annotation and quantitation of expressed loci within additional tumor types or cellular models. One such approach recapitulates ERV transcripts by genome-guided de novo assembly of an ‘LTR transcriptome’ [166]. The analysis of the TCGA LTR transcriptome of 31 cancer types reveals the inclusion of just 17.3% of genomic ERV loci (of 630,356 in GRCh38), of which 3.2% are present in tumor-specific transcripts [166]. ERVs that populate recurrent cancer-specific transcripts (CSTs) represent broad ERV group members but account for less than 1% of annotated loci, implying that the involvement of most ERVs is limited by the cellular environment controlling their expression [166]. For example, the HERV-K 22q11.23 lncRNA *PCAT14* is highly expressed in prostate tumors but also in tumors of the testes and lungs, suggesting accessibility of the locus over multiple tumor types [166]. Many transcripts are associated with ERVL-MaLRs (e.g., older MLT1s, primate-specific MSTs, and simian primate-specific THE1s [58]); young LTR7b and LTR7y HERV-H members, as well as human-specific and unfixed HERV-K HML-2, are also present. Importantly, these findings hint at the limitation in such studies that unannotated insertionally polymorphic LTR5H members may contribute to the data but not be mapped in genome-guided analyses [64]. The variable presence of insertions within relatively new genomic contexts could have profoundly disruptive consequences. Although not a direct comparison, it should be noted that HML-2 proviral expression is biased to older members in normal tissues of GTEx RNAseq; among the ones expressed is the LTR5Hs-driven 22q11.23 *PCAT14* [157]. Thus, highly expressed cancer-specific ERVs represent a relatively small proportion of LTRs, indicating common shifts in the cellular environment between some involved loci.

The landscape of LTR-associated transcripts in cancers is highly complex but is beginning to be disentangled. Mapping of the TCGA cancer-specific transcripts reveals that standalone ERVs account for 17% of the transcripts and LTR-initiated chimeras with gene or lncRNA sequence for 9% [166]. Particularly, LTRs of these latter chimeras provide prime candidates for novel ‘onco-exaptation’ events, in which the reactivation of a LTR drives the overexpression of a proto-oncogene or oncogenic isoform [6,102]. A growing number of examples of LTRs involved in onco-exaptation have been reported [6,29,32,102,103,104,105] and recently reviewed in [106]. For example, a LTR7y/HERV-H cryptic promoter-driven *SLCO1B3* oncogene transcript previously identified in colon, lung, and pancreatic cancers is highly abundant in TCGA of the stomach and esophagus [106,166]. A recent study confirmed KLF5-mediated activation of a LTR7y/HERV-H drives a *CALB1* isoform in lung squamous cell carcinoma [103]. Interestingly, distinct LTRs may also influence the activation of the same gene, possibly due to different cellular contexts. For example, recent studies independently found a MER21B-*E2F3* chimeric transcript among oncogenic transcripts in bladder cancer cell lines [105], whereas a HERV9 LTR12C-*E2F3* transcript is among the top oncogenic transcripts in ovary, prostate, and urothelial cancers [102]. In this latter study, the authors identified 129 TE onco-exaptation events involving 106 genes across 3864 tumors, with at least one event in around 50% of the tumors; onco-exaptation of ERVs was estimated to be one to two-fold higher than other TE classes [102]. Additional ERV-oncogene transcripts include a MaLR MLT1J-*SALL4* predominantly in breast carcinomas and MaLR THE1A-*HMGA2* nearly exclusive to skin cutaneous melanomas [102]. Numerous non-LTR retroelement onco-exaptation events have been reported [102,105].

### 5.4. ERVs Induce a State of ‘Viral Mimicry’

The induction of IFN-stimulated genes (ISGs) is observed in many tumors and cell models and is due to the phenomenon of ‘viral mimicry’ [131]. In viral mimicry, dsRNAs sourced from retroelements (e.g., from bidirectional transcription of a single element, hybridization of transcripts of high sequence similarity, or hairpin structures of inverted repeats) are sensed by the cell, interpreted as a viral infection, and trigger antiviral IFN signaling, setting into action the innate immune response [167,168]. dsRNAs formed via the transcription of inverted repeat SINE/Alu elements appear to be the major driver of viral mimicry activation [169], though LINE, as well as ERV dsRNA species, also trigger an antiviral state [131]. Because the outcomes of this response can include PKR-mediated cell death and increased processing and presentation of TE-derived peptides as tumor-associated antigens, therapeutic agents that expose such immune vulnerabilities of tumors are of high interest, and recent studies have improved our understanding of ERV involvement [131]. For example, induced hypomethylation by the DNMTi decitabine in clear cell renal carcinoma cell lines induces broadly activated ERV groups and antiviral signaling; RNAs of the highest expressed ERVs (e.g., ERV-Fc2-related) are sensor-bound, and the signaling is attenuated by the knockout of MDA5, RIGI, or downstream MAVS [170]. In another study, treatment of pancreatic ductal adenocarcinoma cells with the MEK inhibitor trametinib induced ERV1 (e.g., MERs), ERV-K (including HML-2), and ERV-L (e.g., MLT1s), resulting in a robust MAVS-dependent IFN response [171]. Remarkably, a subset of IFNγ-inducible LTRs (e.g., mostly ERVL-MaLR MLT1, MST members) situated antisense in the 3′ UTRs of several STAT1-inducible genes (e.g., *TNFRSF9*, *TRIM22*, and *TRIM38*) has even evolved to be uniquely primed for bidirectional transcription; they are normally silenced by EZH2, and its knockdown drives a feedforward IFNγ signaling strongly associated with MHC-1 presentation [172]. Candidate ERV loci for contributing to dsRNAs via bidirectional transcription are identified in the TCGA LTR transcriptome; around 30% of highly expressed tumor-specific transcripts possess a terminal LTR, as well as conventional gene TSS [166]. Chromatin regulators have been characterized in the context of ERV-associated viral mimicry [131,173,174]. A regulator of SETDB1 maintenance, PHF8, has been identified as a mediator of tumor immune escape; its ablation stimulates antiviral mimicry in colorectal cancer cells, resulting in the inhibition of tumor growth and immune susceptibility [175]. Consistent with these findings, overexpression of chromatin regulators as SETDB1 and members of the HUSH and TRIM28 complexes are implicated in tumor immune inhibition [131,176]. Depletion of the KZFPs ZNF417 and ZNF587 (primate-specific repressive TFs of evolutionarily young HERV-K [177]) in cells derived from diffuse B-cell lymphoma results in heterochromatin remodeling and IFN signaling, thus enhancing immune susceptibilities [178].

It is important to recognize that cancer cells can likewise adapt to retroelement-driven viral mimicry to circumvent activation of the antiviral state. For example, ADAR1-mediated A-to-I editing of SINE/Alu-derived dsRNAs renders them unrecognizable to the dsRNA sensor MDA5; recent work has demonstrated that ADAR1-dependent cancer cells evade viral mimicry activation, and its depletion reduces tumor growth in patient-derived cancer cells [169]. Systematically screening for viral mimicry adaptations has identified additional proteins involved in cancer dependencies [179]. For example, the RNA decay protein XRN1, which degrades uncapped RNAs (e.g., such as those sourced from transcription of SINE/Alu), confers a dependency in a subset of cancer cell lines; its knockout is associated with reduced cell viability consistent with the induction of viral mimicry [179]. Other cellular proteins in pathways involving RNA modification and nucleic acid metabolism pathways were implicated in the same study [179]. Thus, targeted therapies capable of disrupting such cancer dependencies offer the potential to overcome viral mimicry adaptation, warranting further investigation. Augmenting the antiviral response via ERV activation should represent novel avenues of cancer therapeutics. In this regard, two of the 13 top genes reported alongside XRN1 as regulating viral mimicry adaptation are also present in the TCGA highly expressed tumor-specific LTR transcriptome predicted protein-coding transcripts: *CFLAR* (LTR5Hs-associated in testis) and *ILK* (MalR MLT1M-associated in several tumors) [166,179].

Because IFNs stimulate ISG immune responses involving the antigen presentation machinery, ERV sequences spliced or embedded within transcripts have the potential to produce completely novel antigenic peptides [121,133]. A significant revelation has been that ERVs associated with transcripts in somatic tissues, including tumors, frequently originate from alternate promoters rather than the LTRs themselves [166]. The contextual placement of the ERVs thus needs to be fully considered in RNAseq callsets, as their presence does not necessitate direct use as a promoter or enhancer. For example, chimeras with gene or lncRNA or transcripts with spliced or embedded ERV sequence account for roughly 40% of TCGA cancer-specific highly expressed transcripts [166]. Similar observations have been made in the examination of healthy tissues of GTEx RNAseq for HERV-K HML-2, in which just nine of 37 expressed proviruses had clear 5′ LTR TSSs [157]. In that study, ERV expression by the mechanism of readthrough was epitomized by transcription through a largely truncated LTR5B at 6p25.1 that lacked a 5′ end [157]. The production of immunogenic ERV-derived peptides in an antitumor adaptive response implies the potential for antitumor therapeutic relevance [121,133]. Highly predictable ERV-overlapping transcripts should thus potentially aid in prognosis and understanding cancer-specific antigenicity [166].

### 5.5. ERVs Expressed in Cancers Include Ones Exapted in Development

Several placental genes have been previously identified to possess exapted LTR promoters [95], and a recent work has characterized genes with exapted LTRs that bear enhancer activities in tissues of the placenta [180]. Interestingly, TCGA ERV-associated cancer-specific transcripts overlap genes with exapted LTRs that bear promoter activities in the trophoblast, including *NOS3* (exapted LTR10A promoter), *PTN* (LTR2B/HERV-E), and *HSD17B1* (MER21A) [95,166,181]. These transcripts are present in multiple tumor types and include sequences of the gene and its corresponding LTR. Other genes with reported trophoblast LTR exaptation, for example, the X-linked *MID1* (exapted HERV-E promoter), *ENTPD1* (MER39B), and *ACKR2* (MER39) [181,182], are present in TCGA but associated with alternate LTRs [166]. The exapted *ERVWE1 env*, syncytin-1, is also highly expressed in some TCGA tumors [166]. Many recent studies have implicated the relevance of lncRNAs in various cellular processes [81,82,83,84,85], including in tissues of the trophoblast and placenta; the biological activities of these lncRNAs were recently reviewed in [183]. Notably, TCGA highly expressed tumor-specific transcripts also include ones that overlap with the reported lncRNAs of the trophoblast [166,183]. These include the previously characterized primate-specific LTR7/HERV-H lncRNA *UCA1* that has been recently implicated in the proliferation of human trophoblast stem cells [184], as well as the lncRNAs *SH3PXD2A-AS1*, *RPAIN*, *PROX1*, *MEG3*, and *PVT1* [183]. Deregulation of these lncRNAs is significantly associated with progression in a variety of cancers, as well as early-onset preeclampsia [183]. Similarities have been drawn between developmental tissues such as embryo and trophoblast with cancer cells [185,186]. Possibly, the combination of activated common signaling pathways, as well as a permissible chromatin state, is reflective of the exaptation of ERVs in early development that are susceptible to later reemergence in the cancer landscape [181]. An alternative proposal is that the activation of early developmental LTRs may promote dedifferentiation through the onco-exaptation of genes that influence chromatin states reminiscent of early development, though causative links between the two are not yet clear [187].

## 6. ERV Expression in Embryogenesis Is Precisely Regulated

Recent studies have highlighted the regulation and roles of ERV activation in early cellular development. After fertilization, the genome is in a globally demethylated state [188], and chromatin remodeling is established gradually [189]. The onset of transcription, i.e., zygotic or embryo genome activation (here, EGA), can be characterized by the cell number of the embryo (e.g., two-cell is ‘2C’). EGA varies between mice and humans, widely reported at 2C and by 8C stages, respectively, and ERVs are expressed at each stage [190,191], though recent investigations have revealed earlier low-level transcription in both species, including ERVs [192,193]. Regardless, a clear fact is that precisely regulated lineage-specific ERV expression and subsequent silencing coincides strongly and specifically in a stage-dependent manner in mice and humans, suggesting key roles in species-specific developmental programs [190,194,195]. For example, in mice, MERV-L and ERVL-MalR members are activated in 2C and 4C embryos, whereas ERV-K members are later expressed in the 8C and morula [196]. In humans, studies have shown that HERV-K14 and HERV9 transcripts are present in the oocyte and dramatically increased in the 2C and 8C stages, respectively; HERV-L, ERVL-MaLR, and HERV-H (LTR7b) are expressed in the 8C; HERV-K (LTR5Hs) in the morula; and HERV-H (LTR7y) in the blastocyst [194]. Recent studies have additionally hinted at the activation of similar retroelement expression in the embryos of other placental mammals, such as cow, pig, and dog [197,198,199]. Understood according to broad classification (e.g., ERV1, ERVL, and ERVL-MalR), these findings underpin paths of comparative research in these models. Collectively, these observations have led to the intriguing proposal that species-specific ERV activation may provide a ‘molecular rheostat’ for the regulation of pluripotency [200]. Specific discussion of ongoing and recent findings for those belonging to the mouse ERV-L and human ERV-L, ERV-H, and ERV-K groups follow.

### 6.1. Mouse ERV-L

Members of the *DUX* (double homeobox; mouse *Dux* and human *DUX4*) TF gene family are among the facilitators of EGA [201,202,203]. Promoters of expressed transcripts in mouse embryos are enriched for the Dux TFBS and include 2C gene promoters, as well as LTRs of MERV-L-related lineages (e.g., MuERV-L and ERVL-MaLR) [57,190,201,202,204]. Recent works have highlighted the complexities of Dux/MERV-L regulatory dynamics. Dux activates MERV-L members at the 2C stage, concomitant with EGA [190,201,204]. MERV-L are silenced upon exiting the 2C stage by H3K9 methyltransferases G9a and GLP [3,205]. Upon activation, MERV-L transcripts contribute to ~3% of polyA RNAs in totipotency and serve as a general marker of the 2C stage and a transient 2C-like state [190,195]. The broad depletion of full-length MERV-L transcripts has been shown to cause lethality, with loss of lineage specification and genomic instability, and MERV-L-depleted embryos retain an accessible chromatin structure and aberrant expression of a subset of 2C genes [206]. A recent study indicated that the rapid silencing of Dux by the exit of the 2C stage is mediated by LINE-1 RNAs in a complex with nucleolin-1 and TRIM28/Kap1 and is linked with rRNA synthesis [207], as well as a Dux-induced feedback loop of TRIM24- and TRIM33-mediated silencing via the *Muridae*-specific Duxbl [208]. The silencing of Dux (and in turn, MERV-L) is also linked with a late-2C surge in cytoplasmic viscosity accompanied by nuclear remodeling and nucleoli maturation [209]. Preventing this state leads to incomplete silencing of Dux/MERV-L and cleavage stage arrest [209]. These findings suggest a requirement of the MERV-L presence and strict regulation in 2C embryos, with a putative role in regulating the switch from totipotency to pluripotency [206].

MERV-L transcripts include spliced 5′ LTR-first exon fusions with coding sequences of nonretroviral origin, indicating the exaptation of LTR promoter functions as a resource for the coordinated expression of genes [57,190]. Interestingly, the LTRs linked to these transcripts appear biased by age, with young ERV groups (e.g., *mus*-specific; MT2s) predominantly represented [57]. Among the expressed MERV-L sequences are a proportion of MERV-L MT2 that encode *gag* ORFs, including ones sourced from *mus*-specific insertions amplified within the last ~10 my [56,210]. A subset retains *gag* ORFs, which have been previously shown to contribute to epsilon virus-like particles of an unusual morphology [210]. In this regard, MERV-L-Gag proteins are also present in early embryos at the mid-2C to 4C stages [206], and virus-like particles are observed in the early embryo in the endoplasmic reticulum [190]. The presence of Gag and OCT4 have been shown to be inversely correlated; in totipotent cells where Gag is high, OCT4 is low, and the opposite is observed in pluripotent cells, despite no changes in mRNA levels of the TF [190]. Linking these observations, a recent study has implicated MERV-L-Gag as a modulator of the TFs OCT4 and Sox2 in early-stage (2C) embryos [211]. The study identified a MERV-L Gag binding partner, the prefoldin complex protein URI, which otherwise binds and protects OCT4 and Sox2 from degradation [211]. In this model, the increase in MERV-L Gag displaces URI from either of the two in the 2C stage, leading to OCT4 and Sox2 degradation [211]. The subsequent decrease in Gag levels confers OCT4 and Sox2 actions and the shift to pluripotency [211]. The findings implicate its potential exapted role as a modulator of cell lineage specification in mice in the transition from totipotency to pluripotency. Importantly, this represents the first reported functional interaction of an ERV protein in mouse embryonic development. In this regard, the Gag of a ~10 my old distantly related MERV-L is well characterized for its exapted use as the restriction factor Fv1 [212].

### 6.2. Human ERV-L

Recent studies have advanced our understanding of HERV-L in early development and drawn parallels and distinctions with MERV-L. During the transition to the 2C stage, HERV-L-related LTRs are broadly derepressed with accessible but inactive promoters [213]. HERV-L and ERVL-MaLR members display a marked induction associated with accessible promoter and enhancer-like regions beginning in the 4C stage that is followed by rapid silencing [194,214]. In contrast to activated MERV-L in mouse embryos, recent works have indicated that activated HERV-L includes relatively older ERV-Ls (e.g., MLT2A1 and MLT2A2) [191,214,215]. Although there are MLT2 groups that predate the human–mouse split, these two HERV-L groups entered the germline of simian primate ancestors ~65–45 mya [215]. Their activation in embryogenesis appears to be conserved among the examined extant species (e.g., human, macaque, and marmoset) [215].

Thousands of MLT2As become accessible during the transition from zygote to the 2C stage; their induction coincides with DUX4 gene activation in the 4C and 8C stages, and activated LTRs are indeed shown to be DUX4-bound [201,215,216]. Mapping of the transcripts reveals TSSs are in the LTRs, further indicating precise regulation [215]. Transcribed LTRs tend to be represented by ‘long’ MLT2A members >200 bp, with splice sites mostly to a sequence that is unannotated or within non-coding exons. Spliced transcripts from humans include ones with sequences from at least 21 protein-coding genes; a single spliced protein-coding transcript (i.e., *SH3BGRL*) is present in humans, macaques, and marmosets [215]. In considering mouse Dux activation of 2C genes as well as MERV-L as discussed above, these findings support distinct evolutionary patterns within DUX, which, despite their divergence, have maintained EGA-associated gene promoter interactions, as well as ERV activation by species (e.g., subfamily specificity of HERV-L and MERV-L LTRs of humans or mice), and experienced shifts in the properties of activated ERVs (e.g., the tendency of older vs. younger, respectively). There are several additional HERV-L MLT2-related groups in humans, but none are activated in embryos in the manner of MLT2A1 and MLT2A2, suggested to be due to the lack of DUX4-binding motifs [215].

As discussed in Section 4, among all TFBSs, those for KZFPs are outliers among the most enriched intersecting ERVs; also identified within the top outliers for TFBS enrichment is DUX4 [93]. Aside from ERV-L MLT2As, DUX4-binding motifs are also pervasive within ERVL-MaLRs (e.g., eutherian MLT1s and primate-specific THE1 and MST groups) and are present in relatively minor subsets of other LTR and TE types [60]. For example, of 63,795 DUX4 motifs predicted in the human genome, nearly two-thirds overlap LTRs, and over one-third overlap ERVL-MaLRs [60]. DUX4 activation in 4C embryos of ERVL-MaLR bidirectional enhancer-like regions significantly alters the chromatin accessibility and appears to contribute to regulatory accessible regions and transcripts of EGA genes [214]. Though the repression of these ERV groups is not fully clear, ZNF-mediated H3K9me3 deposition appears to be stage-specific and act on different ERV groups, for example, ZNF766 and ZNF486 bind ERV-MaLR THE1 and MST members in the 8C stage, whereas the ERV-L examined in the study are H3K9me3-unmarked and likely silenced by other mechanisms [113]. As discussed above, a majority of ‘older’ ERV-L members are reported as unresponsive to DNMTis, as well as H3K9me3 inhibitors [114]. Thus, the mechanisms involving ERV-L regulation remain to be clarified and should benefit from further locus-specific characterization of this group. Due to the common presence and DUX-mediated activation of ERV-L- and ERVL-MaLR-related members in humans and mice, similar functions between the two implies their independent exaptation in both species [5].

Embryonic DUX4-driven HERV-L transcripts consist of a large proportion of MLT2A LTRs with splice donor sites fused with gene sequences [194,215]. DUX4 is strictly silenced in differentiated tissues; its re-expression activates TSSs as alternative drivers of genes and lncRNAs in ERVL and MaLR gene chimeras [217] and is implicated in facioscapulohumeral muscular dystrophy [204]. In cancers, DUX4 re-expression is reported to block IFNγ induction of class I MHC antigen presentation, implicating a property of immune evasion [218], and promotes a metastable early embryonic cell program [219]. A recent examination of some somatic tissues implies that reactivated HERV-L may later serve as functional alternative promoters [215]. For example, MLT2A1 appear to be capable of initiating DUX4-independent synthesis and providing the first exons of bona fide protein-coding transcripts (e.g., *ABCE1*, *GALNT13*, and *COL5A1*) when later reactivated in some examined somatic cell types of humans (but, importantly, not macaque), such as the pineal gland [215]. Further, the canonical start codons of *ABCE1* and *GALNT13* are in exon 2 and thus not interrupted in these transcripts. Based on the TFBS profiles of brain tissues, the authors suggested the TF OTX2 as a candidate activator of the associated MLT2As [215]. On this note, ERV-associated tumor-specific transcripts involving all MLT2 groups are accounted for within the tumor-specific TCGA LTR transcriptome [51,166]. Highly expressed tumor-specific TCGA transcripts include alternate LTR chimeras with *GALNT13* (associated LTR12D) in tumors from the brain and adrenal gland, as well as *COL5A1* (MalR THE1B) in lymph nodes [166].

### 6.3. Human ERV-H

The activation of HERV-H is implicated in early embryo programming and serves as a marker thereof [220,221,222]. HERV-H transcripts contribute to roughly 2% of polyA mRNAs in human embryonic stem cells (hESCs), and their activation promotes the maintenance of pluripotency [223]. They are comprised of LTR-initiated chimeric transcripts, including ones with alternative exons, as well as lncRNAs of biological relevance to pluripotency. For example, the lncRNA *linc-ROR* is proposed as a sponge of regulatory miRNAs for OCT4, Sox2, and NANOG to prevent their degradation [221,223,224]. Highly expressed HERV-H demarcate CTCF cell-specific chromatin shaping by establishing topologically associating domain (TAD) boundaries via DNA loop formation and pluripotent chromatin structure [223,225]. Interestingly, CTCF TAD boundaries are lost upon HERV-H depletion, and the random introduction of HERV-H sequences on chromosomes recapitulates TAD boundary formation independent of CTCF [223,225]. Though broad depletion of HERV-H results in the loss of pluripotency in hESCs, there have been mixed results [221,222,224], as has been noted [72], possibly due to sequence differences in constructs used between studies [72,223]. A recent work correlated the silencing of HERV-H lncRNAs with a candidate modulator preventing dedifferentiation, ZBTB12, a conserved BTB-containing ZFP [200]. ZBTB12 binding and association with SIN3A/HDAC is observed locally for ~70 HERV-H loci and correlates strongly with the silencing of HERV-H lncRNAs (e.g., *linc-ROR* and *ESRG*). The ectopic expression of mouse ZBTB12 recapitulates HERV-H silencing in hESCs; its knockout in mouse epiblast stem cells does not impact ERV expression [200]. The authors suggested a scenario of an acquired silencing function during primate evolution, in which HERV-H members inserted near pre-existing ZBTB12 binding sites were positively selected for control of the exit from pluripotency [200].

The recent sequence-based refinement of HERV-H LTRs permits the curation of subgroup properties of preimplantation embryos [72]. Transcripts originating from HERV-H subgroups are differentially enriched across the embryo stages and predominantly sourced from younger LTR7b, LTR7y, and recently defined LTR7up loci [72,194]. Strong LTR7b activation peaks at the 8C stage during EGA and morula [72,194] and thus overlaps in stage presence with HERV-L MLT2A members [194]; the strong induction of LTR7y overlaps this pattern, and LTR7y transcripts are later significantly elevated in the blastocyst [72,194], and LTR7up1/2 are dramatically induced in the blastocyst [72]. Other LTR7s are differentially expressed in stage-specific patterns to a lesser extent [72,194]. Sequence-based analyses of all 5′ and solo-LTR copies reveals a dynamic history resulting in the gain, loss, and exchange of *cis*-regulatory elements among the subgroups [30,72]. The youngest (e.g., LTR7y and LTR7up) appear to have experienced relatively rapid diversification and are among those most highly expressed in early developmental stages [30,72,194], implying the recent evolutionary innovation of precisely regulated sequences. For example, a LTR7up-specific modification is the acquisition of a predicted SOX2/3 TFBS shown in vitro to be necessary for transcription [72]. Many LTR7up loci distinctly overlap with actively bound TFBSs, including ones in the early embryo stages, such as KLF4, NANOG, SOX2, OCT4, and others, in which their sequences are differentially enriched compared to non-transcribed copies and ones of related subgroups [72,221,222], consistent with the TFBS presence from ENCODE and Roadmap Epigenomics data discussed above in Section 3 [86]. However, TF occupancy alone does not fully explain the patterns of transcribed vs. non-transcribed loci [72]. Thus, the observed patterns of HERV-H activation are due, at least in part, to intrinsic LTR properties. Further disentanglement of the properties of activated loci should benefit from the refined characterization of this ERV group and permit targeted analyses by subgroup-specific features.

As mentioned, HERV-H is notable for its pronounced shift in abundance of proviral to solo-LTR copies relative to other HERV groups [28,32,72,226]. This state could reflect HERV-H as a relatively benign component of the genome (e.g., loss of *env* reminiscent of *mus*-specific MERV-L [210]) but is also suggestive of selection on sequences beyond the LTR [31,32]. In this regard, most HERV-H 5′ internal sequences are retained (including three partial *gag* ORFs [39]), and a subset of these proviral loci are positively correlated with transcription in preimplantation embryos, suggestive of selection [32]. Though the mechanisms driving HERV-H preservation are not fully clear, these observations seem to suggest selection in favor of the proviral sequence, for some copies may result from their activities in embryogenesis [31]. The ability to tightly control HERV-H repression while selecting for the internal sequence could be a factor. In this regard, KZFPs (e.g., ZNF534 and ZNF90), as well as KAP1 and H3K9me3 loading, are captured at HERV-H LTR7up1/2 in ChIPseq of hESCs, but neither is clearly enriched nor depleted compared to other HERV-H LTRs, indicating that the repressive actions of these KZFPs do not fully correlate with their regulation in ESCs, thus implicating the involvement of other factors [72].

### 6.4. Human ERV-K

Expressed HERV-K have been reported over the early embryo stages [194,213]. HERV-K HML-1 was active in the ancestors of OWMs ~40–30 mya. Transcripts from HML-1 members (e.g., LTR14B) are present in minor but detectable levels in the oocyte and peak in the 2C stage, returning to minor levels in the blastocyst [194,213]. Another activated HERV-K group is from human-specific HML-2 members (e.g., LTR5Hs), also with a minor presence over multiple stages that peaks in the morula and is considered to be a marker of pluripotency [194,195]. For example, beyond the 8C into the morula, LTR5Hs are decorated with H3K27ac enhancer marks and strongly driven by pluripotency TFs before being rapidly silenced by KZFPs [116]. LTR5H activation promotes open chromatin enhancer states, and experimentally forced repression alters the regulation of genes within <100 kb [116]. Over the past several years, there have been advances to the knowledge of this group.

Among the transcripts from both HERV-K groups are ones attributed to transcription into a flanking sequence with little evidence of splicing [194]. Possibly, some of these contribute to the reported HERV-K Gag-associated particles of the blastocyst [195] (around 17 *gag* ORFs are accounted for over these proviruses [54]), but these observations have yet to be substantiated. Transcripts corresponding to *rec*, an alternatively spliced product of HERVK, have also been reported in the blastocyst stage [195]. Interestingly, the Rec protein appears to associate with and facilitate the transport of nonretroviral mRNAs to the cytoplasm in those cells [195]. Interestingly, overexpression of Rec enhances the IFITM1 mRNA levels, a phenotype that may reflect immunoprotection by an early antiviral response of the embryo [195]. An OCT4-binding motif is present among LTR5Hs (but not older LTR5A nor B); LTRs of expressed LTR5Hs are indeed bound and transactivated by OCT4, and its knockdown depletes LTR5Hs transcripts in early-stage embryos [195]. Thus, HERV-K subgroups appear to harbor sequence-specific functional differences in a regulatory capacity. Consistent with this notion, an analysis of publicly available ChIP-seq data of naïve and primed human ESCs indicated OCT4 and H3K27ac enrichments at LTR5Hs in the former but not the latter, suggesting their activity is also specific to the cell type [227]. Analysis of RNAseq data from the same respective samples revealed the expression of genes up to 120kb from LTR5Hs loci (but not LTR5A nor B), suggestive of long-range enhancer effects. The expression of members of this ERV group should be of keen interest, given its properties as the only known recently active HERV, promoter activities, and coding capacity [64]. These studies should also benefit from assessment of the allelic presence of insertionally polymorphic members, given their functional potential and inferred capability to potentially generate new viruses through recombination [64,68].

### 6.5. The Evolution of DUX Incorporates Species-Specific ERV Activation

It is worth revisiting the case of the DUX TF homologs for the ability to interact with conventional gene promoters, as well as those of LTRs. Dux and DUX4 (mouse and human, respectively) are intronless retroposed homologs originally derived from processed mRNAs of an ancestral *DUX* gene, *DUXC* [228,229,230]. Dux and DUX4 later expanded within macrosatellite arrays in both mice and humans; the intron-containing ancestor was subsequently lost from both species, but its homologs are retained in arrays in Laurasiatherian models (e.g., dog, swine, and bovine), as well as Xenartha (e.g., sloth) [228]. Afrotheria (e.g., elephant, hyrax, and tenrec) possess intronless arrayed homologs from an independently retroposed *DUX* ancestor [228]. These findings place a double homeobox ancestor in placental eutherians ~110 mya and highlight the complex *DUX* evolution within the species’ lineages [228]. A single homeobox *DUX* ancestor is present in amphibians, reptiles, and non-eutherian mammals [228].

As discussed, human DUX4 activates human EGA genes, as well as LTRs belonging to HERV-L [201,204]. A functional analysis of human DUX4 expressed in mouse embryos revealed the activation of common 2C-like orthologous gene promoters but not MERV-L [204]. Of note, both homologs also appear to activate some ERVL-MaLR in the same background, but these are reported to be mostly distinct subsets of elements (<4% in common including just one common alternate promoter) [204]. Intriguingly, canine DUXC expressed in a cultured dog cell model has recently been shown to activate common mouse 2C gene homologs, as well as LTRs of broadly classified ERV groups (e.g., ERV1, ERVL, and ERVL-MaLR MLT1), though the subgroup specificity of the expression is not yet clear [199]. As with human DUX4, canine DUXC expressed in mouse embryos results in the activation of 2C-like gene promoters but not MERV-L [204]. Together, these observations suggest that *DUX* homologs have maintained conserved properties of the transcriptional regulation of gene promoters but have evolved distinct association with LTRs that may be attributed to the divergence of binding within DUX homologs and across species. For example, species-level comparisons of sequence targets and analyses of the protein structure reveals that, despite sharing high structural similarity between the two homeodomains, DUX homeodomain 1 and 2 exhibit different target DNA preferences [231]. For the case of ERV-L subgroups of humans and mice, one function of Dux appears to be involved in the species-specific activation of exapted LTRs, with probable roles in genome activation and/or early cell fate specification. To our knowledge, DUXC transcriptional regulation of retroelements in dogs has not been further explored. The genome of the domestic dog has a relatively low representation of ERVs [18,232] but appears to have retained regulatory properties common to ones expressed in genome activation in humans and mice [42,198,199]. Given the observations in human and mouse DUX-derived functions, and the identified properties of DUXC, it will be interesting to see how the evolutionary history and activation of ERVs by DUXC plays out.

Of relevant note, the retroposed origination and repeated expansion of Dux and DUX4 have been suggested to have been driven by pressure to avoid the activation of propagating retroviruses at the time while maintaining early-stage gene control, reminiscent of mutational escape in an ‘arms race’, as previously proposed to explain KZFP evolution [201]. Such a scenario might account for the divergence of LTR recognition by DUX members but does not explain the species-specific retained functions exerted in regulating ERVs, as is evidenced from the examination of DUX homolog-mediated ERV activation between mice, humans, and dogs. We speculate the alternative scenario in which *DUX* expansion instead took advantage of the ability to activate propagated LTRs and domesticated their use in species-specific embryogenesis regulatory networks.

## 7. Concluding Remarks

The layers of evolved complexity regarding the once-reputed ‘junk’ of our genomes are both astonishing and humbling. As inferred from the ERV fossil record, the scale of virus–host co-evolution stretches a span reaching over 450 million years. The emergence of ERV-repressive KZFPs exemplifies an early established interplay between virus and host and speaks to the importance of wielding ERVs as a functional resource in the subsequent shaping and diversification of genomic landscapes. Alongside this co-evolution between virus and host, the propagation of ERV lineages and *DUX* homolog expansion is reminiscent of a similar scenario of the exploitation of ERVs for bona fide functions, rather than to escape ERV activation in a strict ‘arms race’. The co-evolutionary outcomes are truly remarkable. Our genomes have commandeered ERVs for key roles in many biological processes and are controlled for individual functions (e.g., syncytins), the expression of broad group members (e.g., viral mimicry), and lineage-specific regulation (e.g., immune signaling and early cellular development). The mechanisms contributing to ERV transcriptional control are being disentangled, but layers of complexity undoubtedly remain. ERVs that are tightly regulated during early development can later unleash alternate promoters and enhancers of proto-oncogenes upon the loss of control. The aberrant expression of ERVs in epigenetically altered environments appears to involve a relatively limited number of ERVs compared to those genome-wide but reflect a high degree of heterogeneity in the expressed lineages and subgroups. Conversely, early development appears to control the expression of specific ERV lineages in a highly regulated manner in what seems to be a theme of placental mammals. Fuller annotations of ERV-associated transcripts should provide further insight into their involvement in these and other cellular environments. While the properties of ERVs continue to be more understood in these diverse biological contexts, it is important to keep in mind that many expressed ERV groups are still not well characterized. In this regard, understanding the properties of all involved ERV groups should significantly aid in their future study. Given the range and depth of orthogonal technologies now in use to interrogate the genome and its much accumulated but once coined ‘junk’, it is a truly exciting time for what lies in store.

## References

[1] Wells J.N., Feschotte C. (2020). A Field Guide to Eukaryotic Transposable Elements. Annu. Rev. Genet..

[2] Johnson W.E. (2019). Origins and evolutionary consequences of ancient endogenous retroviruses. Nat. Rev. Microbiol..

[3] Geis F.K., Goff S.P. (2020). Silencing and Transcriptional Regulation of Endogenous Retroviruses: An Overview. Viruses.

[4] Chuong E.B., Elde N.C., Feschotte C. (2016). Regulatory evolution of innate immunity through co-option of endogenous retroviruses. Science.

[5] Chuong E.B., Elde N.C., Feschotte C. (2017). Regulatory activities of transposable elements: From conflicts to benefits. Nat. Rev. Genet..

[6] Babaian A., Mager D.L. (2016). Endogenous retroviral promoter exaptation in human cancer. Mob. DNA.

[7] Kitsou K., Lagiou P., Magiorkinis G. (2023). Human endogenous retroviruses in cancer: Oncogenesis mechanisms and clinical implications. J. Med. Virol..

[8] Aiewsakun P., Katzourakis A. (2017). Marine origin of retroviruses in the early Palaeozoic Era. Nat. Commun..

[9] Anai Y., Ochi H., Watanabe S., Nakagawa S., Kawamura M., Gojobori T., Nishigaki K. (2012). Infectious endogenous retroviruses in cats and emergence of recombinant viruses. J. Virol..

[10] Hayward A., Cornwallis C.K., Jern P. (2015). Pan-vertebrate comparative genomics unmasks retrovirus macroevolution. Proc. Natl. Acad. Sci. USA.

[11] Katzourakis A., Gifford R.J., Tristem M., Gilbert M.T., Pybus O.G. (2009). Macroevolution of complex retroviruses. Science.

[12] Brown P.O., Coffin J.M., Hughes S.H., Varmus H. (1997). Integration. Retroviruses.

[13] Jern P., Coffin J.M. (2008). Effects of retroviruses on host genome function. Annu. Rev. Genet..

[14] Vogt V.M., Coffin J.M., Hughes S.H., Varmus H. (1997). Retroviral Virions and Genomes. Retroviruses.

[15] Stoye J.P. (2012). Studies of endogenous retroviruses reveal a continuing evolutionary saga. Nat. Rev. Microbiol..

[16] Ngo M.H., Arnal M., Sumi R., Kawasaki J., Miyake A., Grant C.K., Otoi T., Fernandez de Luco D., Nishigaki K. (2019). Tracking the Fate of Endogenous Retrovirus Segregation in Wild and Domestic Cats. J. Virol..

[17] Shimode S., Nakagawa S., Miyazawa T. (2015). Multiple invasions of an infectious retrovirus in cat genomes. Sci. Rep..

[18] Halo J.V., Pendleton A.L., Jarosz A.S., Gifford R.J., Day M.L., Kidd J.M. (2019). Origin and recent expansion of an endogenous gammaretroviral lineage in domestic and wild canids. Retrovirology.

[19] Jarosz A.S., Pendleton A.L., Lashbrook M.J., Cech E., Altieri M., Kunch A., Modiano J.F., Halo J.V. (2023). Expression and high levels of insertional polymorphism of an endogenous gammaretrovirus lineage in dogs. PLoS Genet..

[20] Fabryova H., Hron T., Kabickova H., Poss M., Elleder D. (2015). Induction and characterization of a replication competent cervid endogenous gammaretrovirus (CrERV) from mule deer cells. Virology.

[21] Yang L., Malhotra R., Chikhi R., Elleder D., Kaiser T., Rong J., Medvedev P., Poss M. (2021). Recombination Marks the Evolutionary Dynamics of a Recently Endogenized Retrovirus. Mol. Biol. Evol..

[22] Tang L., Swedlund B., Dupont S., Harland C., Costa Monteiro Moreira G., Durkin K., Artesi M., Mullaart E., Sartelet A., Karim L. (2024). GWAS reveals determinants of mobilization rate and dynamics of an active endogenous retrovirus of cattle. Nat. Commun..

[23] Blyton M.D.J., Young P.R., Moore B.D., Chappell K.J. (2022). Geographic patterns of koala retrovirus genetic diversity, endogenization, and subtype distributions. Proc. Natl. Acad. Sci. USA.

[24] Lillie M., Pettersson M., Jern P. (2024). Contrasting segregation patterns among endogenous retroviruses across the koala population. Commun. Biol..

[25] Jern P., Greenwood A.D. (2024). Wildlife endogenous retroviruses: Colonization, consequences, and cooption. Trends Genet..

[26] Hughes J.F., Coffin J.M. (2004). Human endogenous retrovirus K solo-LTR formation and insertional polymorphisms: Implications for human and viral evolution. Proc. Natl. Acad. Sci. USA.

[27] Belshaw R., Watson J., Katzourakis A., Howe A., Woolven-Allen J., Burt A., Tristem M. (2007). Rate of recombinational deletion among human endogenous retroviruses. J. Virol..

[28] Thomas J., Perron H., Feschotte C. (2018). Variation in proviral content among human genomes mediated by LTR recombination. Mob. DNA.

[29] Thompson P.J., Macfarlan T.S., Lorincz M.C. (2016). Long Terminal Repeats: From Parasitic Elements to Building Blocks of the Transcriptional Regulatory Repertoire. Mol. Cell.

[30] Gemmell P., Hein J., Katzourakis A. (2015). Orthologous endogenous retroviruses exhibit directional selection since the chimp-human split. Retrovirology.

[31] Gemmell P., Hein J., Katzourakis A. (2016). Phylogenetic Analysis Reveals That ERVs “Die Young” but HERV-H Is Unusually Conserved. PLoS Comput. Biol..

[32] Gemmell P., Hein J., Katzourakis A. (2019). The Exaptation of HERV-H: Evolutionary Analyses Reveal the Genomic Features of Highly Transcribed Elements. Front. Immunol..

[33] Izsvak Z., Wang J., Singh M., Mager D.L., Hurst L.D. (2016). Pluripotency and the endogenous retrovirus HERVH: Conflict or serendipity?. Bioessays.

[34] Hughes J.F., Coffin J.M. (2001). Evidence for genomic rearrangements mediated by human endogenous retroviruses during primate evolution. Nat. Genet..

[35] Gu W., Zhang F., Lupski J.R. (2008). Mechanisms for human genomic rearrangements. Pathogenetics.

[36] Hughes J.F., Coffin J.M. (2005). Human endogenous retroviral elements as indicators of ectopic recombination events in the primate genome. Genetics.

[37] Trombetta B., Fantini G., D’Atanasio E., Sellitto D., Cruciani F. (2016). Evidence of extensive non-allelic gene conversion among LTR elements in the human genome. Sci. Rep..

[38] Kjeldbjerg A.L., Villesen P., Aagaard L., Pedersen F.S. (2008). Gene conversion and purifying selection of a placenta-specific ERV-V envelope gene during simian evolution. BMC Evol. Biol..

[39] Boso G., Fleck K., Carley S., Liu Q., Buckler-White A., Kozak C.A. (2021). The Oldest Co-opted gag Gene of a Human Endogenous Retrovirus Shows Placenta-Specific Expression and Is Upregulated in Diffuse Large B-Cell Lymphomas. Mol. Biol. Evol..

[40] Mouse Genome Sequencing C., Waterston R.H., Lindblad-Toh K., Birney E., Rogers J., Abril J.F., Agarwal P., Agarwala R., Ainscough R., Alexandersson M. (2002). Initial sequencing and comparative analysis of the mouse genome. Nature.

[41] Venter J.C., Adams M.D., Myers E.W., Li P.W., Mural R.J., Sutton G.G., Smith H.O., Yandell M., Evans C.A., Holt R.A. (2001). The sequence of the human genome. Science.

[42] Lindblad-Toh K., Wade C.M., Mikkelsen T.S., Karlsson E.K., Jaffe D.B., Kamal M., Clamp M., Chang J.L., Kulbokas E.J., Zody M.C. (2005). Genome sequence, comparative analysis and haplotype structure of the domestic dog. Nature.

[43] Pontius J.U., Mullikin J.C., Smith D.R., Agencourt Sequencing T., Lindblad-Toh K., Gnerre S., Clamp M., Chang J., Stephens R., Neelam B. (2007). Initial sequence and comparative analysis of the cat genome. Genome Res..

[44] Nelson P.N., Carnegie P.R., Martin J., Davari Ejtehadi H., Hooley P., Roden D., Rowland-Jones S., Warren P., Astley J., Murray P.G. (2003). Demystified. Human endogenous retroviruses. Mol. Pathol..

[45] Boeke J.D., Stoye J.P., Coffin J., Hughes S., Varmus H. (1997). Retrotransposons, endogenous retroviruses, and the evolution of retroelements. Retroviruses.

[46] Meyer T.J., Rosenkrantz J.L., Carbone L., Chavez S.L. (2017). Endogenous Retroviruses: With Us and against Us. Front. Chem..

[47] Gifford R.J., Blomberg J., Coffin J.M., Fan H., Heidmann T., Mayer J., Stoye J., Tristem M., Johnson W.E. (2018). Nomenclature for endogenous retrovirus (ERV) loci. Retrovirology.

[48] Krupovic M., Blomberg J., Coffin J.M., Dasgupta I., Fan H., Geering A.D., Gifford R., Harrach B., Hull R., Johnson W. (2018). Ortervirales: New Virus Order Unifying Five Families of Reverse-Transcribing Viruses. J. Virol..

[49] Coffin J., Blomberg J., Fan H., Gifford R., Hatziioannou T., Lindemann D., Mayer J., Stoye J., Tristem M., Johnson W. (2021). ICTV Virus Taxonomy Profile: Retroviridae 2021. J. Gen. Virol..

[50] Johnson W.E. (2015). Endogenous Retroviruses in the Genomics Era. Annu. Rev. Virol..

[51] Kojima K.K. (2018). Human transposable elements in Repbase: Genomic footprints from fish to humans. Mob. DNA.

[52] Vargiu L., Rodriguez-Tome P., Sperber G.O., Cadeddu M., Grandi N., Blikstad V., Tramontano E., Blomberg J. (2016). Classification and characterization of human endogenous retroviruses; mosaic forms are common. Retrovirology.

[53] Grandi N., Cadeddu M., Blomberg J., Tramontano E. (2016). Contribution of type W human endogenous retroviruses to the human genome: Characterization of HERV-W proviral insertions and processed pseudogenes. Retrovirology.

[54] Subramanian R.P., Wildschutte J.H., Russo C., Coffin J.M. (2011). Identification, characterization, and comparative genomic distribution of the HERV-K (HML-2) group of human endogenous retroviruses. Retrovirology.

[55] Benit L., Lallemand J.B., Casella J.F., Philippe H., Heidmann T. (1999). ERV-L elements: A family of endogenous retrovirus-like elements active throughout the evolution of mammals. J. Virol..

[56] Costas J. (2003). Molecular characterization of the recent intragenomic spread of the murine endogenous retrovirus MuERV-L. J. Mol. Evol..

[57] Franke V., Ganesh S., Karlic R., Malik R., Pasulka J., Horvat F., Kuzman M., Fulka H., Cernohorska M., Urbanova J. (2017). Long terminal repeats power evolution of genes and gene expression programs in mammalian oocytes and zygotes. Genome Res..

[58] Zuo Z. (2023). The successive emergence of ERVL-MaLRs in primates. Virus Evol..

[59] Magiorkinis G., Belshaw R., Katzourakis A. (2013). ‘There and back again’: Revisiting the pathophysiological roles of human endogenous retroviruses in the post-genomic era. Philos. Trans. R. Soc. Lond. B Biol. Sci..

[60] Young J.M., Whiddon J.L., Yao Z., Kasinathan B., Snider L., Geng L.N., Balog J., Tawil R., van der Maarel S.M., Tapscott S.J. (2013). DUX4 binding to retroelements creates promoters that are active in FSHD muscle and testis. PLoS Genet..

[61] Shi M., Chen F., Sahu S.K., Wang Q., Yang S., Wang Z., Chen J., Liu H., Hou Z., Fang S.G. (2024). Haplotype-resolved chromosome-scale genomes of the Asian and African Savannah Elephants. Sci. Data.

[62] Springer M.S., Murphy W.J., Eizirik E., O’Brien S.J. (2003). Placental mammal diversification and the Cretaceous-Tertiary boundary. Proc. Natl. Acad. Sci. USA.

[63] Lavie L., Medstrand P., Schempp W., Meese E., Mayer J. (2004). Human endogenous retrovirus family HERV-K(HML-5): Status, evolution, and reconstruction of an ancient betaretrovirus in the human genome. J. Virol..

[64] Wildschutte J.H., Williams Z.H., Montesion M., Subramanian R.P., Kidd J.M., Coffin J.M. (2016). Discovery of unfixed endogenous retrovirus insertions in diverse human populations. Proc. Natl. Acad. Sci. USA.

[65] Macfarlane C.M., Badge R.M. (2015). Genome-wide amplification of proviral sequences reveals new polymorphic HERV-K(HML-2) proviruses in humans and chimpanzees that are absent from genome assemblies. Retrovirology.

[66] Holloway J.R., Williams Z.H., Freeman M.M., Bulow U., Coffin J.M. (2019). Gorillas have been infected with the HERV-K (HML-2) endogenous retrovirus much more recently than humans and chimpanzees. Proc. Natl. Acad. Sci. USA.

[67] Turner G., Barbulescu M., Su M., Jensen-Seaman M.I., Kidd K.K., Lenz J. (2001). Insertional polymorphisms of full-length endogenous retroviruses in humans. Curr. Biol..

[68] Dewannieux M., Harper F., Richaud A., Letzelter C., Ribet D., Pierron G., Heidmann T. (2006). Identification of an infectious progenitor for the multiple-copy HERV-K human endogenous retroelements. Genome Res..

[69] Lee Y.N., Bieniasz P.D. (2007). Reconstitution of an infectious human endogenous retrovirus. PLoS Pathog..

[70] Goodchild N.L., Wilkinson D.A., Mager D.L. (1993). Recent evolutionary expansion of a subfamily of RTVL-H human endogenous retrovirus-like elements. Virology.

[71] Jern P. (2005). Genomic Variation and Evolution of HERV-H and Other Endogenous Retroviruses (ERVs). Ph.D. Thesis.

[72] Carter T.A., Singh M., Dumbovic G., Chobirko J.D., Rinn J.L., Feschotte C. (2022). Mosaic cis-regulatory evolution drives transcriptional partitioning of HERVH endogenous retrovirus in the human embryo. Elife.

[73] Costas J. (2002). Characterization of the intragenomic spread of the human endogenous retrovirus family HERV-W. Mol. Biol. Evol..

[74] Perron H., Garson J.A., Bedin F., Beseme F., Paranhos-Baccala G., Komurian-Pradel F., Mallet F., Tuke P.W., Voisset C., Blond J.L. (1997). Molecular identification of a novel retrovirus repeatedly isolated from patients with multiple sclerosis. The Collaborative Research Group on Multiple Sclerosis. Proc. Natl. Acad. Sci. USA.

[75] Perron H., Hamdani N., Faucard R., Lajnef M., Jamain S., Daban-Huard C., Sarrazin S., LeGuen E., Houenou J., Delavest M. (2012). Molecular characteristics of Human Endogenous Retrovirus type-W in schizophrenia and bipolar disorder. Transl. Psychiatry.

[76] Dupressoir A., Lavialle C., Heidmann T. (2012). From ancestral infectious retroviruses to bona fide cellular genes: Role of the captured syncytins in placentation. Placenta.

[77] Imakawa K., Kusama K., Kaneko-Ishino T., Nakagawa S., Kitao K., Miyazawa T., Ishino F. (2022). Endogenous Retroviruses and Placental Evolution, Development, and Diversity. Cells.

[78] Sinha A., Johnson W.E. (2017). Retroviruses of the RDR superinfection interference group: Ancient origins and broad host distribution of a promiscuous Env gene. Curr. Opin. Virol..

[79] Kang B.K., Jung Y.T. (2024). A Replication-Competent Retroviral Vector Expressing the HERV-W Envelope Glycoprotein is a Potential Tool for Cancer Gene Therapy. J. Microbiol. Biotechnol..

[80] Kitao K., Nakagawa S., Miyazawa T. (2021). An ancient retroviral RNA element hidden in mammalian genomes and its involvement in co-opted retroviral gene regulation. Retrovirology.

[81] Fort V., Khelifi G., Hussein S.M.I. (2021). Long non-coding RNAs and transposable elements: A functional relationship. Biochim. Biophys. Acta Mol. Cell Res..

[82] Gibb E.A., Warren R.L., Wilson G.W., Brown S.D., Robertson G.A., Morin G.B., Holt R.A. (2015). Activation of an endogenous retrovirus-associated long non-coding RNA in human adenocarcinoma. Genome Med..

[83] Hu T., Pi W., Zhu X., Yu M., Ha H., Shi H., Choi J.H., Tuan D. (2017). Long non-coding RNAs transcribed by ERV-9 LTR retrotransposon act in cis to modulate long-range LTR enhancer function. Nucleic Acids Res..

[84] Karttunen K., Patel D., Xia J., Fei L., Palin K., Aaltonen L., Sahu B. (2023). Transposable elements as tissue-specific enhancers in cancers of endodermal lineage. Nat. Commun..

[85] Zhou B., Qi F., Wu F., Nie H., Song Y., Shao L., Han J., Wu Z., Saiyin H., Wei G. (2019). Endogenous Retrovirus-Derived Long Noncoding RNA Enhances Innate Immune Responses via Derepressing RELA Expression. mBio.

[86] Ito J., Sugimoto R., Nakaoka H., Yamada S., Kimura T., Hayano T., Inoue I. (2017). Systematic identification and characterization of regulatory elements derived from human endogenous retroviruses. PLoS Genet..

[87] Rabson A.B., Graves B.J., Coffin J.M., Hughes S.H., Varmus H. (1997). Synthesis and Processing of Viral RNA. Retroviruses.

[88] Benachenhou F., Jern P., Oja M., Sperber G., Blikstad V., Somervuo P., Kaski S., Blomberg J. (2009). Evolutionary conservation of orthoretroviral long terminal repeats (LTRs) and ab initio detection of single LTRs in genomic data. PLoS ONE.

[89] Benachenhou F., Sperber G.O., Bongcam-Rudloff E., Andersson G., Boeke J.D., Blomberg J. (2013). Conserved structure and inferred evolutionary history of long terminal repeats (LTRs). Mob. DNA.

[90] Nikitin D., Garazha A., Sorokin M., Penzar D., Tkachev V., Markov A., Gaifullin N., Borger P., Poltorak A., Buzdin A. (2019). Retroelement-Linked Transcription Factor Binding Patterns Point to Quickly Developing Molecular Pathways in Human Evolution. Cells.

[91] Zhou W., Liang G., Molloy P.L., Jones P.A. (2020). DNA methylation enables transposable element-driven genome expansion. Proc. Natl. Acad. Sci. USA.

[92] Consortium E.P., Moore J.E., Purcaro M.J., Pratt H.E., Epstein C.B., Shoresh N., Adrian J., Kawli T., Davis C.A., Dobin A. (2020). Expanded encyclopaedias of DNA elements in the human and mouse genomes. Nature.

[93] Andrews G., Fan K., Pratt H.E., Phalke N., Zoonomia Consortium section s., Karlsson E.K., Lindblad-Toh K., Gazal S., Moore J.E., Weng Z. (2023). Mammalian evolution of human cis-regulatory elements and transcription factor binding sites. Science.

[94] Montesion M., Williams Z.H., Subramanian R.P., Kuperwasser C., Coffin J.M. (2018). Promoter expression of HERV-K (HML-2) provirus-derived sequences is related to LTR sequence variation and polymorphic transcription factor binding sites. Retrovirology.

[95] Cohen C.J., Lock W.M., Mager D.L. (2009). Endogenous retroviral LTRs as promoters for human genes: A critical assessment. Gene.

[96] Grandi N., Erbi M.C., Scognamiglio S., Tramontano E. (2023). Human Endogenous Retrovirus (HERV) Transcriptome Is Dynamically Modulated during SARS-CoV-2 Infection and Allows Discrimination of COVID-19 Clinical Stages. Microbiol. Spectr..

[97] Richardson S.R., Doucet A.J., Kopera H.C., Moldovan J.B., Garcia-Perez J.L., Moran J.V. (2015). The Influence of LINE-1 and SINE Retrotransposons on Mammalian Genomes. Microbiol. Spectr..

[98] Oomen M.E., Torres-Padilla M.E. (2024). Jump-starting life: Balancing transposable element co-option and genome integrity in the developing mammalian embryo. EMBO Rep..

[99] Chuong E.B., Rumi M.A., Soares M.J., Baker J.C. (2013). Endogenous retroviruses function as species-specific enhancer elements in the placenta. Nat. Genet..

[100] Hansen T.J., Fong S.L., Day J.K., Capra J.A., Hodges E. (2024). Human gene regulatory evolution is driven by the divergence of regulatory element function in both cis and trans. Cell Genom..

[101] Hossain M.J., Nyame P., Monde K. (2024). Species-Specific Transcription Factors Associated with Long Terminal Repeat Promoters of Endogenous Retroviruses: A Comprehensive Review. Biomolecules.

[102] Jang H.S., Shah N.M., Du A.Y., Dailey Z.Z., Pehrsson E.C., Godoy P.M., Zhang D., Li D., Xing X., Kim S. (2019). Transposable elements drive widespread expression of oncogenes in human cancers. Nat. Genet..

[103] Attig J., Pape J., Doglio L., Kazachenka A., Ottina E., Young G.R., Enfield K.S., Aramburu I.V., Ng K.W., Faulkner N. (2023). Human endogenous retrovirus onco-exaptation counters cancer cell senescence through calbindin. J. Clin. Investig..

[104] Babaian A., Romanish M.T., Gagnier L., Kuo L.Y., Karimi M.M., Steidl C., Mager D.L. (2016). Onco-exaptation of an endogenous retroviral LTR drives IRF5 expression in Hodgkin lymphoma. Oncogene.

[105] Wang Z., Ying Y., Wang M., Chen Q., Wang Y., Yu X., He W., Li J., Zeng S., Xu C. (2023). Comprehensive identification of onco-exaptation events in bladder cancer cell lines revealed L1PA2-SYT1 as a prognosis-relevant event. iScience.

[106] Zhang M., Zheng S., Liang J.Q. (2022). Transcriptional and reverse transcriptional regulation of host genes by human endogenous retroviruses in cancers. Front. Microbiol..

[107] Groger V., Emmer A., Staege M.S., Cynis H. (2021). Endogenous Retroviruses in Nervous System Disorders. Pharmaceuticals.

[108] Birtle Z., Ponting C.P. (2006). Meisetz and the birth of the KRAB motif. Bioinformatics.

[109] Helleboid P.Y., Heusel M., Duc J., Piot C., Thorball C.W., Coluccio A., Pontis J., Imbeault M., Turelli P., Aebersold R. (2019). The interactome of KRAB zinc finger proteins reveals the evolutionary history of their functional diversification. EMBO J..

[110] Yang P., Wang Y., Macfarlan T.S. (2017). The Role of KRAB-ZFPs in Transposable Element Repression and Mammalian Evolution. Trends Genet..

[111] Yang B.X., El Farran C.A., Guo H.C., Yu T., Fang H.T., Wang H.F., Schlesinger S., Seah Y.F., Goh G.Y., Neo S.P. (2015). Systematic identification of factors for provirus silencing in embryonic stem cells. Cell.

[112] Spencley A.L., Bar S., Swigut T., Flynn R.A., Lee C.H., Chen L.F., Bassik M.C., Wysocka J. (2023). Co-transcriptional genome surveillance by HUSH is coupled to termination machinery. Mol. Cell.

[113] Xu R., Li S., Wu Q., Li C., Jiang M., Guo L., Chen M., Yang L., Dong X., Wang H. (2022). Stage-specific H3K9me3 occupancy ensures retrotransposon silencing in human pre-implantation embryos. Cell Stem Cell.

[114] Ohtani H., Liu M., Zhou W., Liang G., Jones P.A. (2018). Switching roles for DNA and histone methylation depend on evolutionary ages of human endogenous retroviruses. Genome Res..

[115] Imbeault M., Helleboid P.Y., Trono D. (2017). KRAB zinc-finger proteins contribute to the evolution of gene regulatory networks. Nature.

[116] Pontis J., Planet E., Offner S., Turelli P., Duc J., Coudray A., Theunissen T.W., Jaenisch R., Trono D. (2019). Hominoid-Specific Transposable Elements and KZFPs Facilitate Human Embryonic Genome Activation and Control Transcription in Naive Human ESCs. Cell Stem Cell.

[117] de Tribolet-Hardy J., Thorball C.W., Forey R., Planet E., Duc J., Coudray A., Khubieh B., Offner S., Pulver C., Fellay J. (2023). Genetic features and genomic targets of human KRAB-zinc finger proteins. Genome Res..

[118] Turelli P., Playfoot C., Grun D., Raclot C., Pontis J., Coudray A., Thorball C., Duc J., Pankevich E.V., Deplancke B. (2020). Primate-restricted KRAB zinc finger proteins and target retrotransposons control gene expression in human neurons. Sci. Adv..

[119] Playfoot C.J., Duc J., Sheppard S., Dind S., Coudray A., Planet E., Trono D. (2021). Transposable elements and their KZFP controllers are drivers of transcriptional innovation in the developing human brain. Genome Res..

[120] Choudhary M.N.K., Quaid K., Xing X., Schmidt H., Wang T. (2023). Widespread contribution of transposable elements to the rewiring of mammalian 3D genomes. Nat. Commun..

[121] Jansz N., Faulkner G.J. (2021). Endogenous retroviruses in the origins and treatment of cancer. Genome Biol..

[122] Kassiotis G. (2014). Endogenous retroviruses and the development of cancer. J. Immunol..

[123] Costa P., Sales S.L.A., Pinheiro D.P., Pontes L.Q., Maranhao S.S., Pessoa C.D.O., Furtado G.P., Furtado C.L.M. (2023). Epigenetic reprogramming in cancer: From diagnosis to treatment. Front. Cell Dev. Biol..

[124] Mantovani F., Kitsou K., Magiorkinis G. (2024). HERVs: Expression Control Mechanisms and Interactions in Diseases and Human Immunodeficiency Virus Infection. Genes.

[125] Anwar S.L., Wulaningsih W., Lehmann U. (2017). Transposable Elements in Human Cancer: Causes and Consequences of Deregulation. Int. J. Mol. Sci..

[126] Ehrlich M. (2009). DNA hypomethylation in cancer cells. Epigenomics.

[127] Kanholm T., Rentia U., Hadley M., Karlow J.A., Cox O.L., Diab N., Bendall M.L., Dawson T., McDonald J.I., Xie W. (2023). Oncogenic Transformation Drives DNA Methylation Loss and Transcriptional Activation at Transposable Element Loci. Cancer Res..

[128] Montesion M., Bhardwaj N., Williams Z.H., Kuperwasser C., Coffin J.M. (2018). Mechanisms of HERV-K (HML-2) Transcription during Human Mammary Epithelial Cell Transformation. J. Virol..

[129] Patra S.K. (2008). Ras regulation of DNA-methylation and cancer. Exp. Cell Res..

[130] Zhang Q., Pan J., Cong Y., Mao J. (2022). Transcriptional Regulation of Endogenous Retroviruses and Their Misregulation in Human Diseases. Int. J. Mol. Sci..

[131] Chen R., Ishak C.A., De Carvalho D.D. (2021). Endogenous Retroelements and the Viral Mimicry Response in Cancer Therapy and Cellular Homeostasis. Cancer Discov..

[132] Reid Cahn A., Bhardwaj N., Vabret N. (2022). Dark genome, bright ideas: Recent approaches to harness transposable elements in immunotherapies. Cancer Cell.

[133] Yu J., Qiu P., Ai J., Liu B., Han G.Z., Zhu F., Zhang W., Cui J. (2024). Endogenous retrovirus activation: Potential for immunology and clinical applications. Natl. Sci. Rev..

[134] Weiss R.A., Weiss N., Teich H., Varmus H.E., Coffin J. (1984). The search for human RNA tumor viruses. RNA Tumor Viruses.

[135] Callahan R., Drohan W., Tronick S., Schlom J. (1982). Detection and cloning of human DNA sequences related to the mouse mammary tumor virus genome. Proc. Natl. Acad. Sci. USA.

[136] Zhang M., Liang J.Q., Zheng S. (2019). Expressional activation and functional roles of human endogenous retroviruses in cancers. Rev. Med. Virol..

[137] Stricker E., Peckham-Gregory E.C., Scheurer M.E. (2023). CancerHERVdb: Human Endogenous Retrovirus (HERV) Expression Database for Human Cancer Accelerates Studies of the Retrovirome and Predictions for HERV-Based Therapies. J. Virol..

[138] Lee M., Ahmad S.F., Xu J. (2024). Regulation and function of transposable elements in cancer genomes. Cell. Mol. Life Sci..

[139] Liang B., Yan T., Wei H., Zhang D., Li L., Liu Z., Li W., Zhang Y., Jiang N., Meng Q. (2024). HERVK-mediated regulation of neighboring genes: Implications for breast cancer prognosis. Retrovirology.

[140] Aftab A., Shah A.A., Hashmi A.M. (2016). Pathophysiological Role of HERV-W in Schizophrenia. J. Neuropsychiatry Clin. Neurosci..

[141] Tamouza R., Meyer U., Foiselle M., Richard J.R., Wu C.L., Boukouaci W., Le Corvoisier P., Barrau C., Lucas A., Perron H. (2021). Identification of inflammatory subgroups of schizophrenia and bipolar disorder patients with HERV-W ENV antigenemia by unsupervised cluster analysis. Transl. Psychiatry.

[142] Groger V., Cynis H. (2018). Human Endogenous Retroviruses and Their Putative Role in the Development of Autoimmune Disorders Such as Multiple Sclerosis. Front. Microbiol..

[143] Kolbe A.R., Bendall M.L., Pearson A.T., Paul D., Nixon D.F., Perez-Losada M., Crandall K.A. (2020). Human Endogenous Retrovirus Expression Is Associated with Head and Neck Cancer and Differential Survival. Viruses.

[144] Chan S.M., Sapir T., Park S.S., Rual J.F., Contreras-Galindo R., Reiner O., Markovitz D.M. (2019). The HERV-K accessory protein Np9 controls viability and migration of teratocarcinoma cells. PLoS ONE.

[145] Fan J., Qin Z. (2024). Roles of Human Endogenous Retrovirus-K-Encoded Np9 in Human Diseases: A Small Protein with Big Functions. Viruses.

[146] Manca M.A., Solinas T., Simula E.R., Noli M., Ruberto S., Madonia M., Sechi L.A. (2022). HERV-K and HERV-H Env Proteins Induce a Humoral Response in Prostate Cancer Patients. Pathogens.

[147] Grandi N., Tramontano E. (2018). HERV Envelope Proteins: Physiological Role and Pathogenic Potential in Cancer and Autoimmunity. Front. Microbiol..

[148] Skandorff I., Ragonnaud E., Gille J., Andersson A.M., Schrodel S., Duvnjak L., Turner L., Thirion C., Wagner R., Holst P.J. (2023). Human Ad19a/64 HERV-W Vaccines Uncover Immunosuppression Domain-Dependent T-Cell Response Differences in Inbred Mice. Int. J. Mol. Sci..

[149] Lemaitre C., Tsang J., Bireau C., Heidmann T., Dewannieux M. (2017). A human endogenous retrovirus-derived gene that can contribute to oncogenesis by activating the ERK pathway and inducing migration and invasion. PLoS Pathog..

[150] Morozov V.A., Dao Thi V.L., Denner J. (2013). The transmembrane protein of the human endogenous retrovirus--K (HERV-K) modulates cytokine release and gene expression. PLoS ONE.

[151] Kremer D., Gruchot J., Weyers V., Oldemeier L., Gottle P., Healy L., Ho Jang J., Kang T.X.Y., Volsko C., Dutta R. (2019). pHERV-W envelope protein fuels microglial cell-dependent damage of myelinated axons in multiple sclerosis. Proc. Natl. Acad. Sci. USA.

[152] Gruchot J., Lewen I., Dietrich M., Reiche L., Sindi M., Hecker C., Herrero F., Charvet B., Weber-Stadlbauer U., Hartung H.P. (2023). Transgenic expression of the HERV-W envelope protein leads to polarized glial cell populations and a neurodegenerative environment. Proc. Natl. Acad. Sci. USA.

[153] Dittmar T., Hass R. (2023). Intrinsic signalling factors associated with cancer cell-cell fusion. Cell Commun. Signal..

[154] Fei F., Li C., Wang X., Du J., Liu K., Li B., Yao P., Li Y., Zhang S. (2019). Syncytin 1, CD9, and CD47 regulating cell fusion to form PGCCs associated with cAMP/PKA and JNK signaling pathway. Cancer Med..

[155] Li X., Wu X., Li W., Yan Q., Zhou P., Xia Y., Yao W., Zhu F. (2023). HERV-W ENV Induces Innate Immune Activation and Neuronal Apoptosis via linc01930/cGAS Axis in Recent-Onset Schizophrenia. Int. J. Mol. Sci..

[156] Scott E.C., Devine S.E. (2017). The Role of Somatic L1 Retrotransposition in Human Cancers. Viruses.

[157] Burn A., Roy F., Freeman M., Coffin J.M. (2022). Widespread expression of the ancient HERV-K (HML-2) provirus group in normal human tissues. PLoS Biol..

[158] She J., Du M., Xu Z., Jin Y., Li Y., Zhang D., Tao C., Chen J., Wang J., Yang E. (2022). The landscape of hervRNAs transcribed from human endogenous retroviruses across human body sites. Genome Biol..

[159] Garcia-Etxebarria K., Sistiaga-Poveda M., Jugo B.M. (2014). Endogenous retroviruses in domestic animals. Curr. Genom..

[160] Bendall M.L., de Mulder M., Iniguez L.P., Lecanda-Sanchez A., Perez-Losada M., Ostrowski M.A., Jones R.B., Mulder L.C.F., Reyes-Teran G., Crandall K.A. (2019). Telescope: Characterization of the retrotranscriptome by accurate estimation of transposable element expression. PLoS Comput. Biol..

[161] Steiner M.C., Marston J.L., Iniguez L.P., Bendall M.L., Chiappinelli K.B., Nixon D.F., Crandall K.A. (2021). Locus-Specific Characterization of Human Endogenous Retrovirus Expression in Prostate, Breast, and Colon Cancers. Cancer Res..

[162] Shukla S., Zhang X., Niknafs Y.S., Xiao L., Mehra R., Cieslik M., Ross A., Schaeffer E., Malik B., Guo S. (2016). Identification and Validation of PCAT14 as Prognostic Biomarker in Prostate Cancer. Neoplasia.

[163] Bhardwaj N., Montesion M., Roy F., Coffin J.M. (2015). Differential expression of HERV-K (HML-2) proviruses in cells and virions of the teratocarcinoma cell line Tera-1. Viruses.

[164] Houle A.A., Gibling H., Lamaze F.C., Edgington H.A., Soave D., Fave M.J., Agbessi M., Bruat V., Stein L.D., Awadalla P. (2018). Aberrant PRDM9 expression impacts the pan-cancer genomic landscape. Genome Res..

[165] Tomlins S.A., Laxman B., Dhanasekaran S.M., Helgeson B.E., Cao X., Morris D.S., Menon A., Jing X., Cao Q., Han B. (2007). Distinct classes of chromosomal rearrangements create oncogenic ETS gene fusions in prostate cancer. Nature.

[166] Attig J., Young G.R., Hosie L., Perkins D., Encheva-Yokoya V., Stoye J.P., Snijders A.P., Ternette N., Kassiotis G. (2019). LTR retroelement expansion of the human cancer transcriptome and immunopeptidome revealed by de novo transcript assembly. Genome Res..

[167] Chiappinelli K.B., Strissel P.L., Desrichard A., Li H., Henke C., Akman B., Hein A., Rote N.S., Cope L.M., Snyder A. (2016). Inhibiting DNA Methylation Causes an Interferon Response in Cancer via dsRNA Including Endogenous Retroviruses. Cell.

[168] Roulois D., Loo Yau H., Singhania R., Wang Y., Danesh A., Shen S.Y., Han H., Liang G., Jones P.A., Pugh T.J. (2015). DNA-Demethylating Agents Target Colorectal Cancer Cells by Inducing Viral Mimicry by Endogenous Transcripts. Cell.

[169] Mehdipour P., Marhon S.A., Ettayebi I., Chakravarthy A., Hosseini A., Wang Y., de Castro F.A., Loo Yau H., Ishak C., Abelson S. (2020). Epigenetic therapy induces transcription of inverted SINEs and ADAR1 dependency. Nature.

[170] de Cubas A.A., Dunker W., Zaninovich A., Hongo R.A., Bhatia A., Panda A., Beckermann K.E., Bhanot G., Ganesan S., Karijolich J. (2020). DNA hypomethylation promotes transposable element expression and activation of immune signaling in renal cell cancer. JCI Insight.

[171] Cortesi A., Gandolfi F., Arco F., Di Chiaro P., Valli E., Polletti S., Noberini R., Gualdrini F., Attanasio S., Citron F. (2024). Activation of endogenous retroviruses and induction of viral mimicry by MEK1/2 inhibition in pancreatic cancer. Sci. Adv..

[172] Canadas I., Thummalapalli R., Kim J.W., Kitajima S., Jenkins R.W., Christensen C.L., Campisi M., Kuang Y., Zhang Y., Gjini E. (2018). Tumor innate immunity primed by specific interferon-stimulated endogenous retroviruses. Nat. Med..

[173] Guo Y., Mao X., Xiong L., Xia A., You J., Lin G., Wu C., Huang L., Wang Y., Yang S. (2021). Structure-Guided Discovery of a Potent and Selective Cell-Active Inhibitor of SETDB1 Tudor Domain. Angew. Chem. Int. Ed. Engl..

[174] Zanre V., Bellinato F., Cardile A., Passarini C., Monticelli J., Di Bella S., Menegazzi M. (2024). Lamivudine, Doravirine, and Cabotegravir Downregulate the Expression of Human Endogenous Retroviruses (HERVs), Inhibit Cell Growth, and Reduce Invasive Capability in Melanoma Cell Lines. Int. J. Mol. Sci..

[175] Liu Y., Hu L., Wu Z., Yuan K., Hong G., Lian Z., Feng J., Li N., Li D., Wong J. (2023). Loss of PHF8 induces a viral mimicry response by activating endogenous retrotransposons. Nat. Commun..

[176] Griffin G.K., Wu J., Iracheta-Vellve A., Patti J.C., Hsu J., Davis T., Dele-Oni D., Du P.P., Halawi A.G., Ishizuka J.J. (2021). Epigenetic silencing by SETDB1 suppresses tumour intrinsic immunogenicity. Nature.

[177] Yang B., Fang L., Gao Q., Xu C., Xu J., Chen Z.X., Wang Y., Yang P. (2022). Species-specific KRAB-ZFPs function as repressors of retroviruses by targeting PBS regions. Proc. Natl. Acad. Sci. USA.

[178] Martins F., Rosspopoff O., Carlevaro-Fita J., Forey R., Offner S., Planet E., Pulver C., Pak H., Huber F., Michaux J. (2024). A Cluster of Evolutionarily Recent KRAB Zinc Finger Proteins Protects Cancer Cells from Replicative Stress-Induced Inflammation. Cancer Res..

[179] Hosseini A., Lindholm H.T., Chen R., Mehdipour P., Marhon S.A., Ishak C.A., Moore P.C., Classon M., Di Gioacchino A., Greenbaum B. (2024). Retroelement decay by the exonuclease XRN1 is a viral mimicry dependency in cancer. Cell Rep..

[180] Sun M.A., Wolf G., Wang Y., Senft A.D., Ralls S., Jin J., Dunn-Fletcher C.E., Muglia L.J., Macfarlan T.S. (2021). Endogenous Retroviruses Drive Lineage-Specific Regulatory Evolution across Primate and Rodent Placentae. Mol. Biol. Evol..

[181] Frost J.M., Amante S.M., Okae H., Jones E.M., Ashley B., Lewis R.M., Cleal J.K., Caley M.P., Arima T., Maffucci T. (2023). Regulation of human trophoblast gene expression by endogenous retroviruses. Nat. Struct. Mol. Biol..

[182] Landry J.R., Rouhi A., Medstrand P., Mager D.L. (2002). The Opitz syndrome gene Mid1 is transcribed from a human endogenous retroviral promoter. Mol. Biol. Evol..

[183] Adu-Gyamfi E.A., Cheeran E.A., Salamah J., Enabulele D.B., Tahir A., Lee B.K. (2024). Long non-coding RNAs: A summary of their roles in placenta development and pathologydagger. Biol. Reprod..

[184] Kong X., Li R., Chen M., Zheng R., Wang J., Sun C., Qu Y. (2024). Endogenous retrovirus HERVH-derived lncRNA UCA1 controls human trophoblast development. Proc. Natl. Acad. Sci. USA.

[185] Costanzo V., Bardelli A., Siena S., Abrignani S. (2018). Exploring the links between cancer and placenta development. Open Biol..

[186] Smith Z.D., Shi J., Gu H., Donaghey J., Clement K., Cacchiarelli D., Gnirke A., Michor F., Meissner A. (2017). Epigenetic restriction of extraembryonic lineages mirrors the somatic transition to cancer. Nature.

[187] Lynch-Sutherland C.F., Chatterjee A., Stockwell P.A., Eccles M.R., Macaulay E.C. (2020). Reawakening the Developmental Origins of Cancer Through Transposable Elements. Front. Oncol..

[188] Iurlaro M., von Meyenn F., Reik W. (2017). DNA methylation homeostasis in human and mouse development. Curr. Opin. Genet. Dev..

[189] Du Z., Zheng H., Huang B., Ma R., Wu J., Zhang X., He J., Xiang Y., Wang Q., Li Y. (2017). Allelic reprogramming of 3D chromatin architecture during early mammalian development. Nature.

[190] Macfarlan T.S., Gifford W.D., Driscoll S., Lettieri K., Rowe H.M., Bonanomi D., Firth A., Singer O., Trono D., Pfaff S.L. (2012). Embryonic stem cell potency fluctuates with endogenous retrovirus activity. Nature.

[191] Taubenschmid-Stowers J., Rostovskaya M., Santos F., Ljung S., Argelaguet R., Krueger F., Nichols J., Reik W. (2022). 8C-like cells capture the human zygotic genome activation program in vitro. Cell Stem Cell.

[192] Asami M., Lam B.Y.H., Hoffmann M., Suzuki T., Lu X., Yoshida N., Ma M.K., Rainbow K., Guzvic M., VerMilyea M.D. (2023). A program of successive gene expression in mouse one-cell embryos. Cell Rep..

[193] Asami M., Lam B.Y.H., Ma M.K., Rainbow K., Braun S., VerMilyea M.D., Yeo G.S.H., Perry A.C.F. (2022). Human embryonic genome activation initiates at the one-cell stage. Cell Stem Cell.

[194] Goke J., Lu X., Chan Y.S., Ng H.H., Ly L.H., Sachs F., Szczerbinska I. (2015). Dynamic transcription of distinct classes of endogenous retroviral elements marks specific populations of early human embryonic cells. Cell Stem Cell.

[195] Grow E.J., Flynn R.A., Chavez S.L., Bayless N.L., Wossidlo M., Wesche D.J., Martin L., Ware C.B., Blish C.A., Chang H.Y. (2015). Intrinsic retroviral reactivation in human preimplantation embryos and pluripotent cells. Nature.

[196] Zhang W., Chen F., Chen R., Xie D., Yang J., Zhao X., Guo R., Zhang Y., Shen Y., Goke J. (2019). Zscan4c activates endogenous retrovirus MERVL and cleavage embryo genes. Nucleic Acids Res..

[197] Halstead M.M., Ma X., Zhou C., Schultz R.M., Ross P.J. (2020). Chromatin remodeling in bovine embryos indicates species-specific regulation of genome activation. Nat. Commun..

[198] Nip Y., Bennett S.R., Smith A.A., Jones T.I., Jones P.L., Tapscott S.J. (2023). Human DUX4 and porcine DUXC activate similar early embryonic programs in pig muscle cells: Implications for preclinical models of FSHD. Hum. Mol. Genet..

[199] Wong C.J., Whiddon J.L., Langford A.T., Belleville A.E., Tapscott S.J. (2022). Canine DUXC: Implications for DUX4 retrotransposition and preclinical models of FSHD. Hum. Mol. Genet..

[200] Han D., Liu G., Oh Y., Oh S., Yang S., Mandjikian L., Rani N., Almeida M.C., Kosik K.S., Jang J. (2023). ZBTB12 is a molecular barrier to dedifferentiation in human pluripotent stem cells. Nat. Commun..

[201] Hendrickson P.G., Dorais J.A., Grow E.J., Whiddon J.L., Lim J.W., Wike C.L., Weaver B.D., Pflueger C., Emery B.R., Wilcox A.L. (2017). Conserved roles of mouse DUX and human DUX4 in activating cleavage-stage genes and MERVL/HERVL retrotransposons. Nat. Genet..

[202] Ren W., Gao L., Mou Y., Deng W., Hua J., Yang F. (2022). DUX: One Transcription Factor Controls 2-Cell-like Fate. Int. J. Mol. Sci..

[203] Smith C.M., Grow E.J., Shadle S.C., Cairns B.R. (2023). Multiple repeat regions within mouse DUX recruit chromatin regulators to facilitate an embryonic gene expression program. bioRxiv.

[204] Whiddon J.L., Langford A.T., Wong C.J., Zhong J.W., Tapscott S.J. (2017). Conservation and innovation in the DUX4-family gene network. Nat. Genet..

[205] Maksakova I.A., Thompson P.J., Goyal P., Jones S.J., Singh P.B., Karimi M.M., Lorincz M.C. (2013). Distinct roles of KAP1, HP1 and G9a/GLP in silencing of the two-cell-specific retrotransposon MERVL in mouse ES cells. Epigenetics Chromatin.

[206] Sakashita A., Kitano T., Ishizu H., Guo Y., Masuda H., Ariura M., Murano K., Siomi H. (2023). Transcription of MERVL retrotransposons is required for preimplantation embryo development. Nat. Genet..

[207] Xie S.Q., Leeke B.J., Whilding C., Wagner R.T., Garcia-Llagostera F., Low Y., Chammas P., Cheung N.T., Dormann D., McManus M.T. (2022). Nucleolar-based Dux repression is essential for embryonic two-cell stage exit. Genes. Dev..

[208] Vega-Sendino M., Luttmann F.F., Olbrich T., Chen Y., Kuenne C., Stein P., Tillo D., Carey G.I., Zhong J., Savy V. (2024). The homeobox transcription factor DUXBL controls exit from totipotency. Nat. Genet..

[209] Ye Y., Homer H.A. (2024). A surge in cytoplasmic viscosity triggers nuclear remodeling required for Dux silencing and pre-implantation embryo development. Cell Rep..

[210] Ribet D., Louvet-Vallee S., Harper F., de Parseval N., Dewannieux M., Heidmann O., Pierron G., Maro B., Heidmann T. (2008). Murine endogenous retrovirus MuERV-L is the progenitor of the “orphan” epsilon viruslike particles of the early mouse embryo. J. Virol..

[211] de la Rosa S., Del Mar Rigual M., Vargiu P., Ortega S., Djouder N. (2024). Endogenous retroviruses shape pluripotency specification in mouse embryos. Sci. Adv..

[212] Best S., Le Tissier P., Towers G., Stoye J.P. (1996). Positional cloning of the mouse retrovirus restriction gene Fv1. Nature.

[213] Liu L., Leng L., Liu C., Lu C., Yuan Y., Wu L., Gong F., Zhang S., Wei X., Wang M. (2019). An integrated chromatin accessibility and transcriptome landscape of human pre-implantation embryos. Nat. Commun..

[214] Vuoristo S., Bhagat S., Hyden-Granskog C., Yoshihara M., Gawriyski L., Jouhilahti E.M., Ranga V., Tamirat M., Huhtala M., Kirjanov I. (2022). DUX4 is a multifunctional factor priming human embryonic genome activation. iScience.

[215] Hashimoto K., Jouhilahti E.M., Tohonen V., Carninci P., Kere J., Katayama S. (2021). Embryonic LTR retrotransposons supply promoter modules to somatic tissues. Genome Res..

[216] DiRusso J.A., Clark A.T. (2023). Transposable elements in early human embryo development and embryo models. Curr. Opin. Genet. Dev..

[217] Mitsuhashi S., Nakagawa S., Sasaki-Honda M., Sakurai H., Frith M.C., Mitsuhashi H. (2021). Nanopore direct RNA sequencing detects DUX4-activated repeats and isoforms in human muscle cells. Hum. Mol. Genet..

[218] Chew G.L., Campbell A.E., De Neef E., Sutliff N.A., Shadle S.C., Tapscott S.J., Bradley R.K. (2019). DUX4 Suppresses MHC Class I to Promote Cancer Immune Evasion and Resistance to Checkpoint Blockade. Dev. Cell.

[219] Smith A.A., Nip Y., Bennett S.R., Hamm D.C., Lemmers R., van der Vliet P.J., Setty M., van der Maarel S.M., Tapscott S.J. (2023). DUX4 expression in cancer induces a metastable early embryonic totipotent program. Cell Rep..

[220] Santoni F.A., Guerra J., Luban J. (2012). HERV-H RNA is abundant in human embryonic stem cells and a precise marker for pluripotency. Retrovirology.

[221] Wang J., Xie G., Singh M., Ghanbarian A.T., Rasko T., Szvetnik A., Cai H., Besser D., Prigione A., Fuchs N.V. (2014). Primate-specific endogenous retrovirus-driven transcription defines naive-like stem cells. Nature.

[222] Ohnuki M., Tanabe K., Sutou K., Teramoto I., Sawamura Y., Narita M., Nakamura M., Tokunaga Y., Nakamura M., Watanabe A. (2014). Dynamic regulation of human endogenous retroviruses mediates factor-induced reprogramming and differentiation potential. Proc. Natl. Acad. Sci. USA.

[223] Sexton C.E., Tillett R.L., Han M.V. (2022). The essential but enigmatic regulatory role of HERVH in pluripotency. Trends Genet..

[224] Lu X., Sachs F., Ramsay L., Jacques P.E., Goke J., Bourque G., Ng H.H. (2014). The retrovirus HERVH is a long noncoding RNA required for human embryonic stem cell identity. Nat. Struct. Mol. Biol..

[225] Zhang Y., Li T., Preissl S., Amaral M.L., Grinstein J.D., Farah E.N., Destici E., Qiu Y., Hu R., Lee A.Y. (2019). Transcriptionally active HERV-H retrotransposons demarcate topologically associating domains in human pluripotent stem cells. Nat. Genet..

[226] Katzourakis A., Pereira V., Tristem M. (2007). Effects of recombination rate on human endogenous retrovirus fixation and persistence. J. Virol..

[227] Fuentes D.R., Swigut T., Wysocka J. (2018). Systematic perturbation of retroviral LTRs reveals widespread long-range effects on human gene regulation. Elife.

[228] Leidenroth A., Clapp J., Mitchell L.M., Coneyworth D., Dearden F.L., Iannuzzi L., Hewitt J.E. (2012). Evolution of DUX gene macrosatellites in placental mammals. Chromosoma.

[229] Bosnakovski D., Toso E.A., Ener E.T., Gearhart M.D., Yin L., Luttmann F.F., Magli A., Shi K., Kim J., Aihara H. (2023). Antagonism among DUX family members evolved from an ancestral toxic single homeodomain protein. iScience.

[230] Jagannathan S. (2022). The evolution of DUX4 gene regulation and its implication for facioscapulohumeral muscular dystrophy. Biochim. Biophys. Acta Mol. Basis Dis..

[231] Lee J.K., Bosnakovski D., Toso E.A., Dinh T., Banerjee S., Bohl T.E., Shi K., Orellana K., Kyba M., Aihara H. (2018). Crystal Structure of the Double Homeodomain of DUX4 in Complex with DNA. Cell Rep..

[232] Halo J.V., Pendleton A.L., Shen F., Doucet A.J., Derrien T., Hitte C., Kirby L.E., Myers B., Sliwerska E., Emery S. (2021). Long-read assembly of a Great Dane genome highlights the contribution of GC-rich sequence and mobile elements to canine genomes. Proc. Natl. Acad. Sci. USA.

